# Ultrasmall, Coating-Free, Pyramidal Platinum Nanoparticles for High Stability Fuel Cell Oxygen Reduction

**DOI:** 10.1021/acsami.2c07738

**Published:** 2022-08-03

**Authors:** Valentina Mastronardi, Emanuele Magliocca, José Solla Gullon, Rosaria Brescia, Pier Paolo Pompa, Thomas S. Miller, Mauro Moglianetti

**Affiliations:** † Nanobiointeractions & Nanodiagnostics, Istituto Italiano di Tecnologia, Via Morego 30, 16163 Genova, Italy; ‡ Department of Chemistry and Industrial Chemistry, University of Genova, Via Dodecaneso 31, 16146 Genova, Italy; § Electrochemical Innovation Laboratory, Department of Chemical Engineering, University College London, Torrington Place, WC1E 7JE London, U.K.; ∥ Institute of Electrochemistry, University of Alicante, Apdo. 99, E-03080 Alicante, Spain; ⊥ Electron Microscopy Facility, Istituto Italiano di Tecnologia, Via Morego 30, 16163 Genova, Italy

**Keywords:** single-crystal Pt pyramidal nanocatalysts, {111} surface domains, oxygen reduction reaction, full polymer electrolyte membrane fuel cells (PEMFCs), durability, aggregation/corrosion resistance

## Abstract

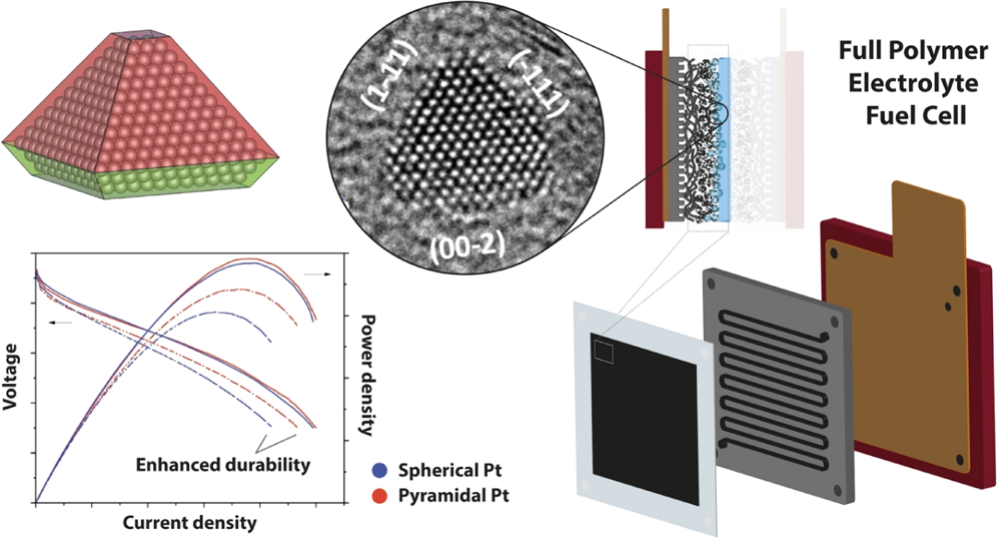

Ultrasmall (<5 nm diameter) noble metal nanoparticles with a high fraction of {111} surface domains are of fundamental and practical interest as electrocatalysts, especially in fuel cells; the nanomaterial surface structure dictates its catalytic properties, including kinetics and stability. However, the synthesis of size-controlled, pure Pt-shaped nanocatalysts has remained a formidable chemical challenge. There is an urgent need for an industrially scalable method for their production. Here, a one-step approach is presented for the preparation of single-crystal pyramidal nanocatalysts with a high fraction of {111} surface domains and a diameter below 4 nm. This is achieved by harnessing the shape-directing effect of citrate molecules, together with the strict control of oxidative etching while avoiding polymers, surfactants, and organic solvents. These catalysts exhibit significantly enhanced durability while, providing equivalent current and power densities to highly optimized commercial Pt/C catalysts at the beginning of life (BOL). This is even the case when they are tested in full polymer electrolyte membrane fuel cells (PEMFCs), as opposed to rotating disk experiments that artificially enhance electrode kinetics and minimize degradation. This demonstrates that the {111} surface domains in pyramidal Pt nanoparticles (as opposed to spherical Pt nanoparticles) can improve aggregation/corrosion resistance in realistic fuel cell conditions, leading to a significant improvement in membrane electrode assembly (MEA) stability and lifetime.

## Introduction

Metallic nanoparticles have been intensively studied as catalytic materials,^1^ and great understanding of their physicochemical properties has been achieved to date, paving the way for their industrial application in a wide range of applications from pharmaceuticals to sensing and energy. Over time, the critical importance of the nanoparticle shape in defining its performance has been recognized; shape dictates the surface arrangement of the atoms, and hence, it strongly modulates and enhances the particle properties from improved selectivity in catalytic processes to tunable interaction with light and living matter.^2−11^ The rate of catalytic activity also depends on the shape and size of nanoparticles, and therefore, the synthesis of well-controlled shapes and sizes of colloidal nanoparticles can significantly improve catalytic performance.^12^ Although several methods to obtain specific surface atomic arrangements of metal nanoparticles have been reported in the literature,^2,13−17^ few have achieved ultrasmall (<5 nm diameter) noble metal nanoparticles without the use of polymers, surfactants, organic solvents, and other directing agents, which are highly difficult to adequately remove after synthesis and are often toxic.^18^ In this framework, the control of the shape of ultrasmall nanoparticles is particularly challenging.

Synthetic strategies to obtain shaped Pt nanocrystals with preferential surface structure mainly work through the stabilization of a facet using molecules that selectively stabilize specific surface domains, influencing the crystal growth along a specific direction and tailoring the final shape of the particle.^19−21^ However, the shape-directing coating often remains bound on the nanocrystal surface and affects, most often deteriorating, the catalytic properties of the material. In particular, it has been clearly established that shape-directing agents such as poly(vinylpyrrolidone) (PVP), tetradecyl trimethyl ammonium bromide, and oleylamine are catalyst poisons.^19,21−25^ Several purification steps have been proposed to remove these organic coatings, but most are time-consuming, costly, do not guarantee complete removal and, importantly, may themselves interfere with the surface structure and catalytic properties of the nanocrystals.^20,21,26^

Another major challenge in the synthesis of surface-tailored catalytic nanoparticles is that, as the particle size decreases, the proportion of specific desired facets (e.g., {111} and {100}) tends to decrease dramatically, while low coordination sites such as edges, steps, corners, and kinks become predominant on the surface.^27^ From a geometric point of view, the area ratio of flat terraces to edges and corners falls for ultrasmall nanomaterials. Consequently, the possibility of designing few-nanometer Pt nanocatalysts with a high percentage of {111} facets is a complex research challenge.

The creation of high-mass-activity and high-stability platinum nanoparticles is a particularly timely challenge because of their role in many vital next-generation clean energy technologies.^28,29^ Platinum catalysts are routinely used in polymer electrolyte membrane fuel cells (PEMFCs) as the kinetics of the oxygen reduction reaction (ORR) at the cathode are otherwise sluggish.^30^

Although the ORR can be catalyzed by non-Pt catalysts in alkaline conditions, acidic proton-exchange systems are the most commercially developed technology and here the performance of Pt catalysts is still largely unsurpassed, certainly at scale.^29^ Hence, due to the high cost of Pt, there is significant need to maximize the utilization and longevity of the catalysts in PEMFC stacks. This is usually achieved through the utilization of small (3–5 nm) Pt nanoparticles supported on carbon black,^29^ commonly formed via a simultaneous reduction/deposition process resulting in polycrystalline Pt structures. However, these catalysts suffer from degradation via corrosion of the carbon support or the aggregation, migration, or dissolution of the Pt nanoparticles.^31^

It is well understood that electrochemical processes proceed at different rates on different Pt crystal planes^32−34^ and hence selecting for a particular facet can boost a particular component of the electrochemical performance. For example, the ORR has been shown to proceed at the highest rate on the Pt {100} surface in H_2_SO_4_,^33^ although this changes depending on the electrolyte.^32^ Similarly, the interaction with Nafion, the primary membrane material for acidic PEMFCs, is dependent on the Pt facet,^35,36^ with {111} showing particular affinity that can impact ORR kinetics. However, ex situ electrochemical tests on Pt(111) surfaces have also been shown to demonstrate significantly superior resistance to surface rearrangement and dissolution.^37,38^ Yet, while this is a crucial property for fuel cells, it has not been shown in realistic full cell conditions with the catalysts in a membrane electrode assembly (MEA).

Enhanced ORR kinetics have also been demonstrated at specific facets of bimetallic catalysts, which help overcome issues related to the high cost and relative scarcity of Pt. In particular, PtNi catalysts have been the object of intensive research since it was shown that the Pt_3_Ni(111) surface is ten times more active for the ORR than the corresponding Pt(111).^39^ In octahedral nanoparticles, such enhancement is maximized as it exclusively exposes a highly active “Pt-skin” structure.^40−42^ Yet, along with this increased activity, other issues accompany PtNi-based catalysts, including catalyst low durability due to Ni dissolution, which is compounded by strong anisotropic elemental distributions on the surface of octahedral PtNi particles.^43^ This raises concerns about their viability for commercial fuel cell applications.

The testing of new catalysts is, in fact, rarely demonstrated in conditions that are representative of those in an operational fuel cell (Table S1). Testing is most commonly limited to rotating disk (RDE)/rotating ring disk experiments, but these are far from representative of the complex environment at the triple phase boundary in a PEMFC MEA. To our knowledge, the application of shape-controlled Pt nanoparticles for the ORR has yet to be demonstrated within an MEA in a full fuel cell,^44,45^ meaning that the true impact of shape control has yet to be understood.^46^

Here, we demonstrate a single-step synthetic procedure to obtain ultrasmall Pt nanocrystals in an aqueous environment, resulting in nanocrystals with an optimal average size of 3.4 nm and a high percentage of dissolution resistant {111} facets. These have been utilized in full PEMFCs and tested using both standardized US Department of Energy (DoE) accelerated stress tests (ASTs) and enhanced ASTs designed to be representative of commercial fuel cell conditions^47,48^ to reveal the true impact of the surface structure on the catalyst and cell durability.

## Results and Discussion

### Synthesis of Pt Nanocatalysts

**Figure 1:**
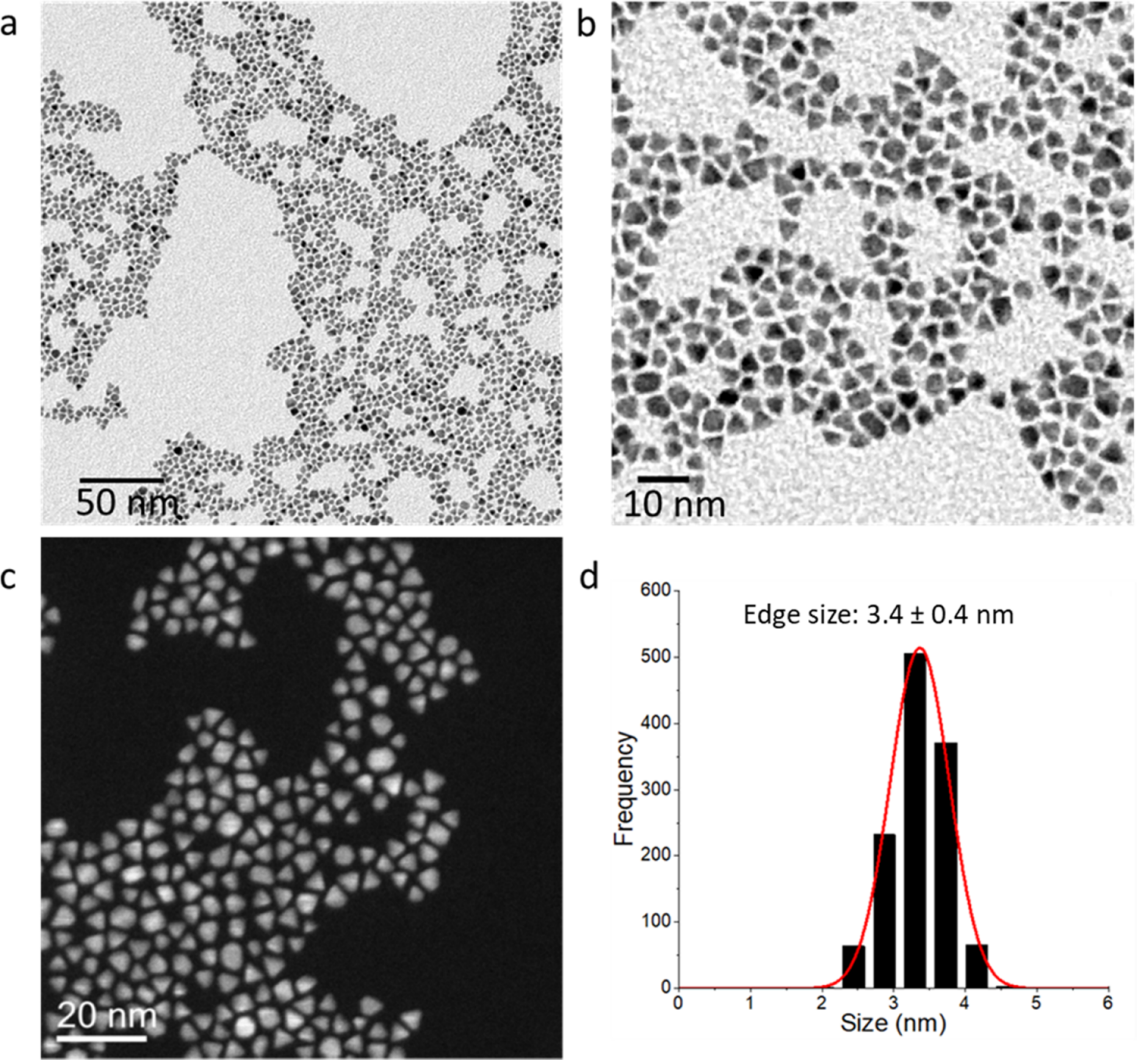
Transmission electron microscopy images of pyramidal platinum nanoparticles. (a, b) Bright-field transmission electron microscopy (BF-TEM) images of pyramidal platinum nanoparticles with high monodispersity in shape and size. (c) High-angle annular dark-field scanning transmission electron microscopy (HAADF-STEM) image showing the monodispersity in the shape of the nanocrystals. (d) Distribution of the nanocrystal sizes.

Ultrasmall (<4 nm diameter) pyramidal Pt nanoparticles with a high fraction of {111} facets were synthesized in an aqueous environment without the use of polymers, surfactants, and other difficult-to-remove capping agents (Figures 1 and S1). A scheme of the process can be seen in Figure S1de. As depicted, this method is fast (only requiring 10 min at 90 °C), easily scalable, and based on the shape-directing ability of sodium citrate molecules. Moreover, it does not require a complex setup as it is simply performed in an airtight capped vessel.

**Figure 2:**
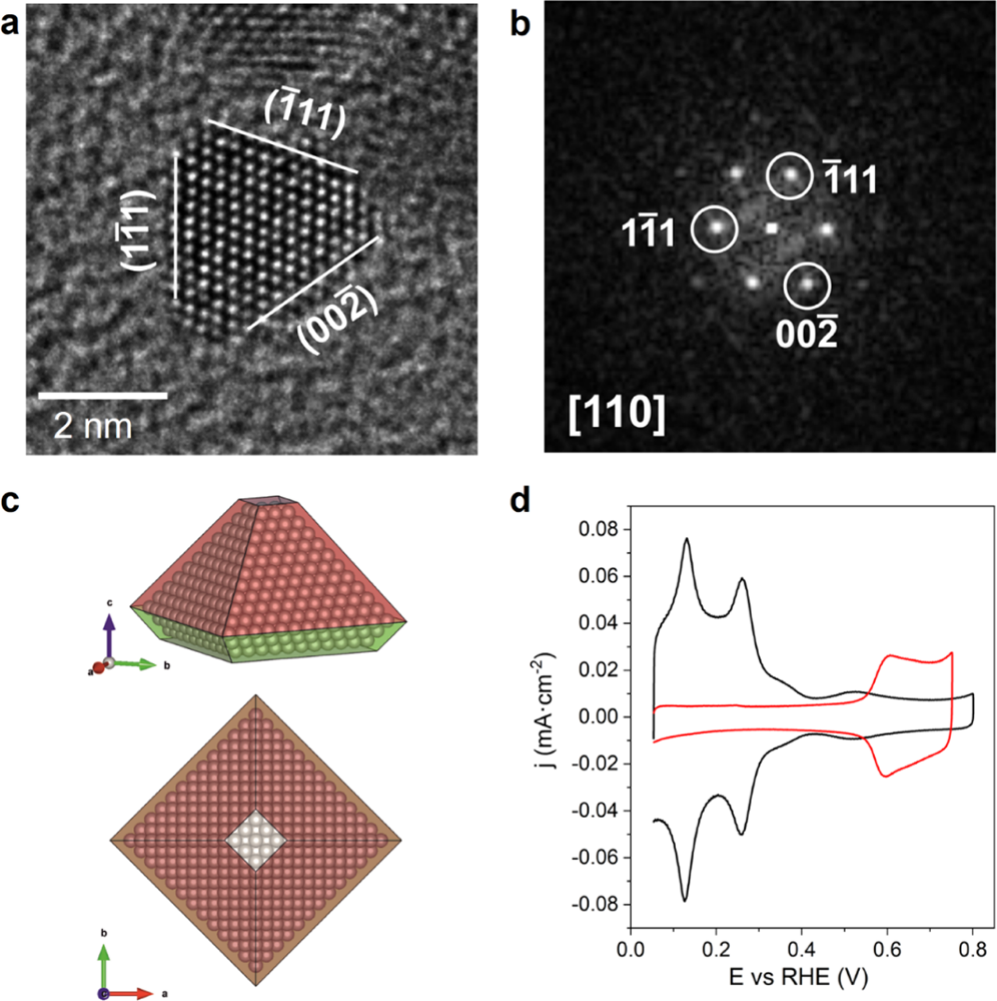
High-resolution transmission electron microscopy (HR-TEM) analysis and electrochemical characterization of pyramidal Pt NPs. (a) HR-TEM image of a pyramidal nanoparticle in the sample and (b) corresponding fast Fourier transform (FFT) pattern, both indexed according to cubic Pt (ICSD 41525). (c) Schematic model of a 3.4 nm base edge slightly truncated pyramidal Pt NP, with a square {001} base and four triangular {111} facets, in agreement with the HR-TEM observations, obtained using VESTA. (d) Cyclic voltammograms corresponding to the ∼3 nm Pt nanoparticles before (black line) and after (red line) Bi adsorption. Test solution: 0.5 M H_2_SO_4_, sweep rate 50 mV s^–1^.

The pyramidal shape of the nanomaterials and the narrow polydispersity clearly emerge from analysis of the bright-field transmission electron microscopy (BF-TEM) images (Figure 1a,b) and high-angle annular dark-field scanning TEM (HAADF-STEM) images (Figure 1c). From the histogram in Figure 1d, it can be seen that the particles’ edge size ranged from 2.5 to 4 nm, with an average of 3.4 (±0.4) nm. On the other hand, Feret’s diameter is equal to 3.9 (±0.6) nm, as shown in Figure S1c. XRD analysis confirmed an average crystallite domain size of around 3 nm, estimated with the use of the Scherrer equation. A high proportion of the nanoparticles has the desired square-based pyramid shape, as shown in Figure 1a–c. Due to this shape control, it is possible to establish the nature of the Pt crystal facets at each surface. The HR-TEM image in Figure 2a shows a typical pyramidal particle, oriented along the most often occurring orientation, the ⟨110⟩ direction of Pt. As already suggested by the thickness contrast in HAADF-STEM images (Figure 1c), the shape of the particles can be described by an octahedron truncated by two {100} planes, one more extended than the other. In this shape, most of the surface is formed of {111} planes.

From a VESTA^49^ schematic model of pyramidal NP with an edge length equal to 3.4 nm, we calculated that almost 50% of the atoms are located on the surface of the nanomaterial, in position to perform catalytic processes. This is important to achieve maximum performance while limiting the amount of precious metal employed in the system.

Here, key roles are played by sodium citrate in combination with sodium borohydride, along with limited oxidative etching, in promoting the synthesis of pyramidal-shaped Pt nanoparticles. Sodium citrate and sodium borohydride play a synergistic role in this process as they have two different reducing potentials that act in combination.^50^ Sodium borohydride promotes the growth of tiny nuclei, while sodium citrate guides the subsequent anisotropic growth. This interplay of strong and weak reducing agents alters the reduction rates, promotes anisotropic growth, and, hence, results in the kinetic growth conditions^4,51^ needed to trap the nanomaterial in a less favorable shape, from a surface energy point of view (Table S2 and Figures S2–S9).

Regarding the role of citrate in favouring anisotropy, Herrero et al.^52^ have recorded, through electrochemical experiments, Fourier transform infrared spectra and established density functional theory that citrate ions in an aqueous solution can become adsorbed on the Pt(111) surface via all three dehydrogenated carboxylic groups in a bidentate configuration. Such a ligand configuration means that citrate is more favorably adsorbed on the Pt(111) surface when compared to the other two Pt basal planes. This is a major advantage in the synthesis of preferentially shaped nanoparticles as it stabilizes the {111} crystallographic facet during the growth phase in solution. However, in contrast to those used in other methods, these ligands can be easily removed by simple washing with a basic aqueous solution. However, sodium citrate alone is not enough to obtain nanoparticles with close to exclusive pyramidal shapes (Table S2 and Figure S9). Only the synergy between sodium citrate and sodium borohydride during synthesis in a closed environment at a relatively low temperature produces ultrasmall nanoparticles with a pyramidal shape.

A pivotal role is also played by the control of the oxidative etching during the growth, which was limited by a closed vessel environment and by working at a relatively low temperature (90 °C). Higher temperatures and high concentrations of oxidants like O_2_ can favor the reshaping of the newly formed nanoparticles and promote the formation of irregular shapes. In the case of an open vessel, the formation of more polydisperse in size and shape nanoparticles has been observed, likely due to the excess of oxygen (Figures S2 and S3). Moreover, citrate molecules can play a role in limiting the effect of oxygen. Indeed, citrate could adsorb onto the surface of newly formed clusters. Once present on the surface, these species can compete with oxygen adsorption and reduce the amount of oxygen immobilized on the surface, while, at the same time, they can react with oxygen and exhaust the adsorbed oxygen.^8^

The absence of impurities in the Pt precursors is also important in promoting anisotropic growth of nanoparticles.^53^ Only with extremely purified Pt precursors (as obtained from producers that, thanks to highly optimized crystallization processes, can guarantee high purity and absence of major contaminants), we obtain the formation of pyramidal shape. The majority of commercially available Pt precursors, if not further purified, favored the synthesis of spherical nanoparticles (Figure S6).

Hence, we can conclude that sodium citrate in combination with sodium borohydride and limited oxidative etching controlled the outcome and the geometry of the Pt nanoparticles. In this way, we can achieve a highly clean surface, a crucial aspect for catalysis, without losing stability and without major aggregation processes. To the best of our knowledge, the goal of ultrasmall, preferentially shaped, Pt nanoparticles has never been accomplished in one-pot water-based, fast, and easily scalable synthesis. Only one recent report has achieved sub-5 nm carbon-supported Pt nanocubes displaying a high percentage of {100} facets, obtained through reshaping of spherical NPs once deposited on carbon support.^54^ This promising report further emphasizes the need to develop simple synthetic methods that control the growth and the shape of the nanomaterials directly in solution to craft the materials on the application requirements.

### Electrochemical Characterization of the Pyramidal Pt NPs

When preparing catalysts, it is important to utilize tools to characterize the “real” surface structure of the nanoparticles. This is, however, complex because of the presence of surface domains of various dimensions and different geometries, as well as a high fraction of surface defects, including corner and edge sites. In addition, the surface structure analysis must also be statistically representative of the sample and should account for the intrinsic heterogeneity of each batch of nanoparticles, as the resulting electrocatalytic activity (for any reaction) will be the sum of the contributions of each type of surface site present. As demonstrated in previous reports,^18,55−57^ some electrochemical probes have been shown to be exceptionally powerful for the in situ study of the surface structure of different types of shaped metal nanoparticles. For Pt nanoparticles, the cyclic voltammetric profile of the so-called “hydrogen region” (where hydrogen and anion adsorption–desorption occurs), obtained in 0.5 M H_2_SO_4_, is a standard way to qualitatively study their surface structure.^18,58^ The response is a “fingerprint” of the Pt surface structure, and, from the relative intensity of the different electrochemical features, it is possible to analyze the surface structure of any Pt surface.

Figure 2d shows a representative voltammetric response of the ultrasmall (∼3 nm) Pt nanocrystals in deaerated 0.5 M H_2_SO_4_. The sharpness and symmetry of the different voltammetric features are indicative of the cleanliness of the surface of the nanoparticles. As discussed in previous contributions,^18,58^ the peak at 0.125 V corresponds to hydrogen adsorption/desorption on Pt(110) sites, whereas the peak at 0.26 V is due to the Pt(100) step sites on Pt(111) terraces and those sites close to the steps on the Pt(100) terraces. It is well established that Pt(100) bidimensional terraces produce a broad contribution close to 0.35–0.37 V, but, as expected from the tetrahedral shape of the nanoparticles, this contribution is very small in these samples. Finally, the main characteristic of the voltammogram is the contribution at 0.5–0.55 V, which is related to the presence of well-defined bidimensionally ordered Pt(111) terraces. This finding qualitatively demonstrates that the ∼3 nm Pt nanocrystals display a preferential {111} surface structure. In fact, the obtained voltammogram closely resembles that previously obtained with octahedral Pt nanocrystals with larger particle sizes,^18^ although it should be noted that the current nanoparticles display lower exclusivity of {111} sites, which would be expected from their smaller particle size. As previously mentioned, a significant decrease in particle size leads to an increase in the presence of corner and edge surface atoms as compared to terraces (additional electrochemical characterization can be found in Figure S10).

To quantitatively analyze the presence of {111} surface domains, Bi adsorption experiments were performed; Bi is known to irreversibly adsorb onto Pt(111) surfaces and the redox processes of this layer can be used to quantify the Pt surface structure.^57,59^ The results are shown in Figure 2d. From the charge involved in the oxidation process at ∼0.60 V and using the relationships deduced for a series of well-defined stepped surfaces,^59^ the percentage of {111} surface domains can be obtained. The analysis indicates that the fraction of {111} domains on the surface of the nanoparticles is close to 21%, namely, a very high proportion for 3 nm nanoparticles, although it is lower than the value previously reported for 7 nm octahedral Pt nanoparticles (39% of {111} domains).^18^

### Deposition of Pyramidal Pt NPs on Amorphous Carbon Supports

**Figure 3:**
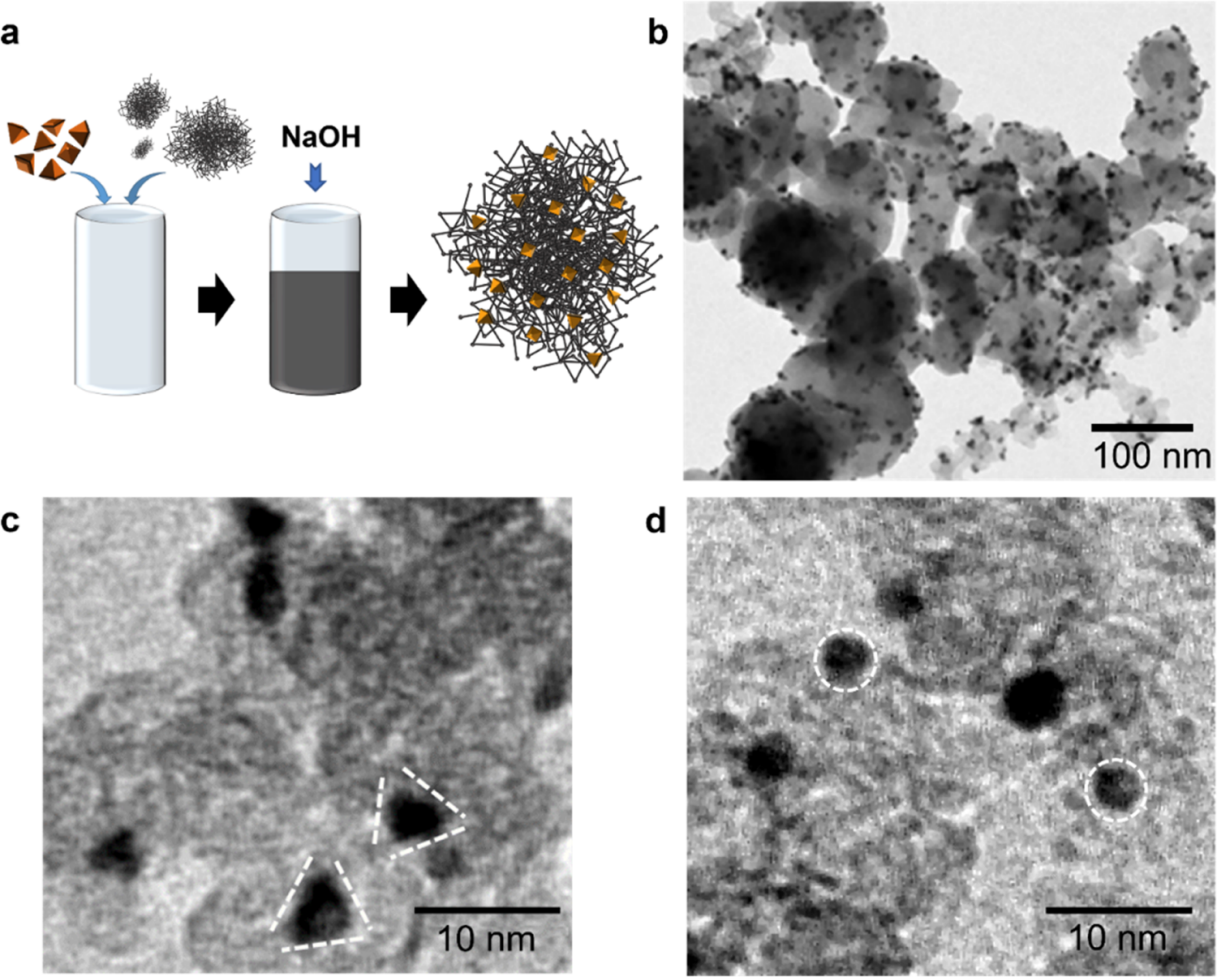
Deposition of pyramidal Pt NPs on an amorphous carbon support. (a) Schematic representation of the simple process for the deposition of pyramidal Pt NPs on amorphous carbon. (b) Morphology of pyramidal Pt/C as visualized by BF-TEM. The BF-TEM image shows the pyramidal Pt NPs in black and the Vulcan support in gray. Higher magnification of pyramidal and spherical Pt/C is shown in (c) and (d), respectively.

The pyramidal Pt NPs were supported on Vulcan carbon black XC72R (Figure 3a). This supported Pt catalyst is henceforth denoted py-Pt/C. The BF-TEM morphology of the pristine, as prepared py-Pt/C is shown in Figure 3b,c. After deposition on carbon (gray regions), the Pt NPs (dark regions) retained their pyramidal shape, as the method of deposition is simple and does not require harsh etching chemicals. The Pt NPs were uniformly distributed on the carbon support and showed no significant agglomeration (consistent with the structures found in commercial Pt/C catalysts). Figures 3d and S11 show equivalent data for spherical Pt nanoparticles prepared using a similar aqueous citrate capped method (sp-Pt/C), whereas Figure S11 shows commercial Pt on carbon.

The citrate coating allows the deposition process of Pt nanopyramids to be simple, effective, and fast. The removal of citrate molecules can be easily achieved by increasing the pH value of the aqueous solution to 10 or above. The change in binding modes of citrate molecules on noble metal surfaces when moving from acidic pH to basic pH has been extensively examined using solid-state NMR, IR, and XPS.^60^ The alkaline conditions convert the monodentate citrate ion into trisodium citrate, which undergoes tetradentate coordination upon binding of the hydroxyl and carboxyl groups to the surface. As a consequence, the citrate molecules repel electrostatically. At high values of pH, these repulsive forces between highly charged citrate molecules on the nanoparticle surface become dominant, leading to detachment of citrate molecules and consequent deposition/precipitation of the NPs on the carbon support. This process is an easy, scalable, and effective deposition strategy, greatly leveraging the advantages of citrate coating and the absence of difficult-to-remove coatings. Indeed, it guarantees the almost complete removal of citrate without provoking any damage to the Pt surface. The mass percentage of Pt pyramidal NPs dispersed on carbon was determined via thermogravimetric analysis to be 21% (Figure S12).

### Performance of py-Pt/C Catalysts in Full Polymer Electrolyte Membrane Fuel Cells

Before full PEMFC tests were performed, the py-Pt/C and sp-Pt/C catalysts were assessed ex situ via RDE experiments in a 0.1 M HClO_4_ electrolyte (Figure S13). Interestingly, the performance of both catalysts was very similar, with low overpotentials consistent with both commercial and literature Pt catalysts.^21,61^ This suggests that using the py-Pt/C catalyst with high {111} density does not in practice negatively impact electrode kinetics significantly, despite the {100} surface being associated with the fastest ORR.^32,33^ The specific activity was also the same at an equivalent loading. After 30 000 AST RDE cycles (performed according to the DoE protocols, see the Materials and Methods section), the ORR activity of both catalysts was largely unchanged. However, the sp-Pt/C was found to have lost 25% of its (electrochemical surface area) ECSA over the 30 000 cycles, whereas the py-Pt/C only lost 13% (Figure S14). This is important as it highlights that the highly favorable mass transport conditions at the RDE can mask catalyst failure modes such as agglomeration, but these changes can have a significant impact on full cell performance,^46,61^ demonstrating why it is important that new catalysts are assessed in full cells.

**Figure 4:**
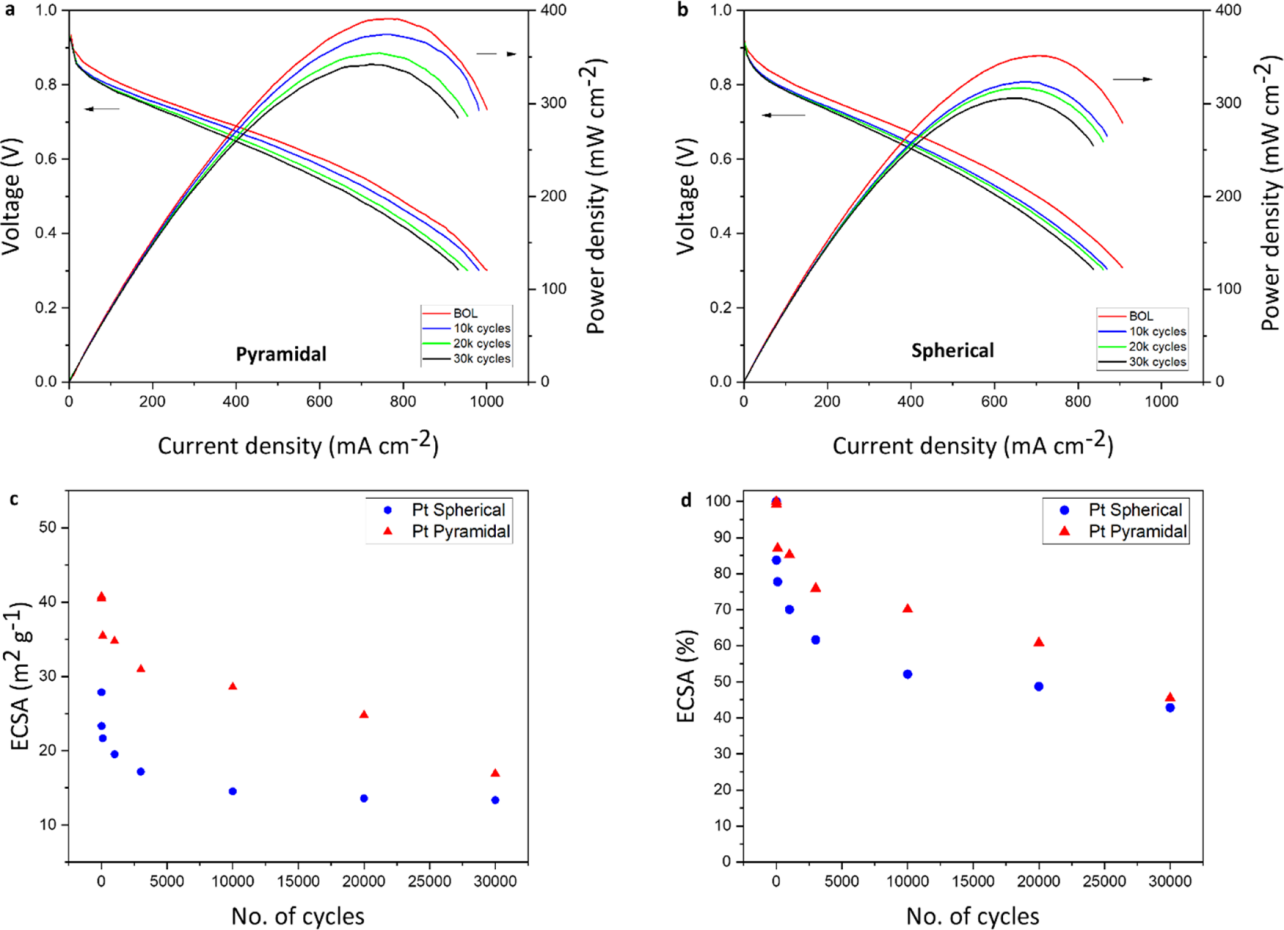
Electrocatalytic fuel cell characterization. Full PEMFC polarization curves at the BOL and after 10–30k AST cycles for (a) pyramidal (py-Pt/C) and (b) spherical (sp-Pt/C) catalysts. Tests were performed using 100% relative humidity H_2_/air at a cell temperature of 80 °C with no back pressure. ECSA values for the two different catalysts (Pt spherical in blue and Pt pyramidal in red) during ASTs are shown in (c) as the absolute change (m^2^ g^–1^) and in (d) as the percentage change (%).

To test the full cell activity of the py-Pt/C compared to other catalysts, single-cell PEMFC experiments were initially conducted using 5 cm^2^ MEAs. All cathodes were prepared via the same process, although with different Pt/C sources, with a consistent geometrical Pt-mass loading of 0.4 mg_Pt_ cm^–2^. The same commercial Pt/C-based anode was used for all experiments (Pt loading: 0.4 mg_Pt_ cm^–2^). Cells were operated under humidified H_2_ and air, with a stoichiometry of 1.5 and 3 for anode and cathode, respectively, at 80 °C and 100% relative humidity without back pressure (more details in the Materials and Methods section). Figure 4a shows the polarization curves for py-Pt/C (as shown in Figure 3b,c), and Figure 4b shows equivalent data for the spherical Pt nanoparticles (sp-Pt/C) (Figures 3d and S11). At the beginning of life (BOL—red line Figure 4a,b), the open-circuit voltage (OCV) is 0.93 and 0.94 V for the sp-Pt/C and py-Pt/C catalyst MEAs, respectively, indicative of effective and properly functioning cells.^62,63^ Beyond their OCV, polarization curves are traditionally analyzed based on their performance in three key regions: in the mass transport (∼0.5 to 0.3 V) and Ohmic (∼0.7 to 0.5 V) regions, both cells behave similarly at the BOL, indicating comparable MEA behavior with regard to the movement of active species and resistance as expected, considering that the particle shape should not impact these factors. This further confirms that the preparation of the catalyst layer and MEA was consistent and uniform for both sp-Pt/C and py-Pt/C catalysts. Most importantly, the polarization curves are also consistent in the kinetic region (OCV – ∼0.7 V) at the BOL, demonstrating good catalyst utilization and turnover for both catalysts. At 0.8 V, the current densities measured are 123 and 126 mA cm^–2^ for the sp-Pt/C and py-Pt/C catalysts, particularly important as there is no significant indication of more sluggish kinetics at the pyramidal particles with a high proportion of {111} facets, as shown in fundamental studies at single-crystal surfaces in aqueous electrolytes.^32−34^ The peak current density for the py-Pt/C catalyst at 0.3 V is 1001 mA cm^–2^, significantly higher than the sp-Pt/C (907 mA cm^–2^), although both are typical for in-house deposited catalyst layers with 5 cm^2^ MEAs. It should be underlined that no back pressure was used in these small geometric area cell experiments, and air was the source of O_2_.^61^ The peak power density at the BOL is similarly significantly higher for the py-Pt/C (391 mW cm^–1^ vs 351 mW cm^–1^ for sp-Pt/C).

The cells were then subjected to ASTs over 30 000 cycles according to the US DoE testing metrics (more details in the Materials and Methods section). Although both cells can be seen to degrade over this period (Figure 4a,b—blue, green and black lines), as would be expected, it can be observed that the sp-Pt/C shows significant performance losses over the first 10 000 cycles (Figure 4b—blue line), while the py-Pt/C material is significantly more stable (Figure 4a—blue line). Importantly, by 10 000 cycles, the pyramidal Pt with a high concentration of {111} facets is the only catalyst to have lost less than 30 mV at 800 mA cm^–2^ (16 mV vs 28 mV for sp-Pt/C), a target set by the US DoE.^48^ This is important as it highlights the fact that MEA conditions are more electrochemically challenging than those usually used to test electrocatalysts, such as RDE.^46^

A comparison of the ECSA of the Pt catalysts as the ASTs proceed, obtained by the hydrogen underpotential deposition method, offers evidence of the expected^37,38^ corrosion resistance of the py-Pt/C (Figure 4c). While the spherical particles prepared using a similar one-pot method (sp-Pt/C—blue circles) lose ECSA sharply over the first ∼3000 cycles, the py-Pt/C (red triangles) drops much more slowly, consistent with the results from RDE, showing that the physicochemical properties these particles have in common beyond shape (easy-to-remove coating, purity, and dispersion on carbon) are not solely responsible for the enhanced stability. Outside of a small initial drop of the ECSA, the change of py-Pt/C is close to linear over time, as can be observed when the percentage of ECSA loss is compared (Figure 4b—red triangles). It can be seen that the py-Pt/C catalyst maintains a significantly higher ECSA, power and current density for the vast majority of its operational lifetime.

To help explain why the pyramidal catalyst system offers enhanced durability, further RDE experiments were performed to assess the morphologies of py-Pt/C and sp-Pt/C before and after AST. Here, each catalyst was supported on a carbon-coated TEM grid so their structure could be assessed at the nanoscale. TEM images (Figures S15 and S16) showed that before testing the catalyst loading and distribution of both catalysts were consistent and equivalent to the samples shown in Figure 3. These same TEM grids were then used as the electrodes for RDE-based ASTs over 30 000 cycles (using a specialized electrode holder, see the Materials and Methods section) so the structural change could be directly assessed in situ, as shown in Figures S17 and S18. While the py-Pt/C catalysts show little observable change in catalyst distribution or loading (Figure S17), a highly significant change can be seen for sp-Pt/C (Figure S18) with large areas of supporting carbon now clear of Pt and large agglomerates of Pt observable elsewhere. This is consistent with the dramatic change in ECSA shown for the spherical catalysts both ex situ (Figure S14c) and in situ (Figure 4c,d) and suggests that the pyramidal Pt is indeed resistant to surface rearrangement and dissolution that can drive agglomeration.^37,38^ This is supported by the fact that the pyramidal shape of the Pt can still be observed for the py-Pt/C catalysts after the 30 000 AST cycles (Figure S19). Importantly, while this type of structural change may not significantly hinder RDE analysis, the challenging mass transport conditions within a full PEMFC MEA mean it is utmost importance to maintain good catalyst distribution and available surface area.^61^

**Figure 5:**
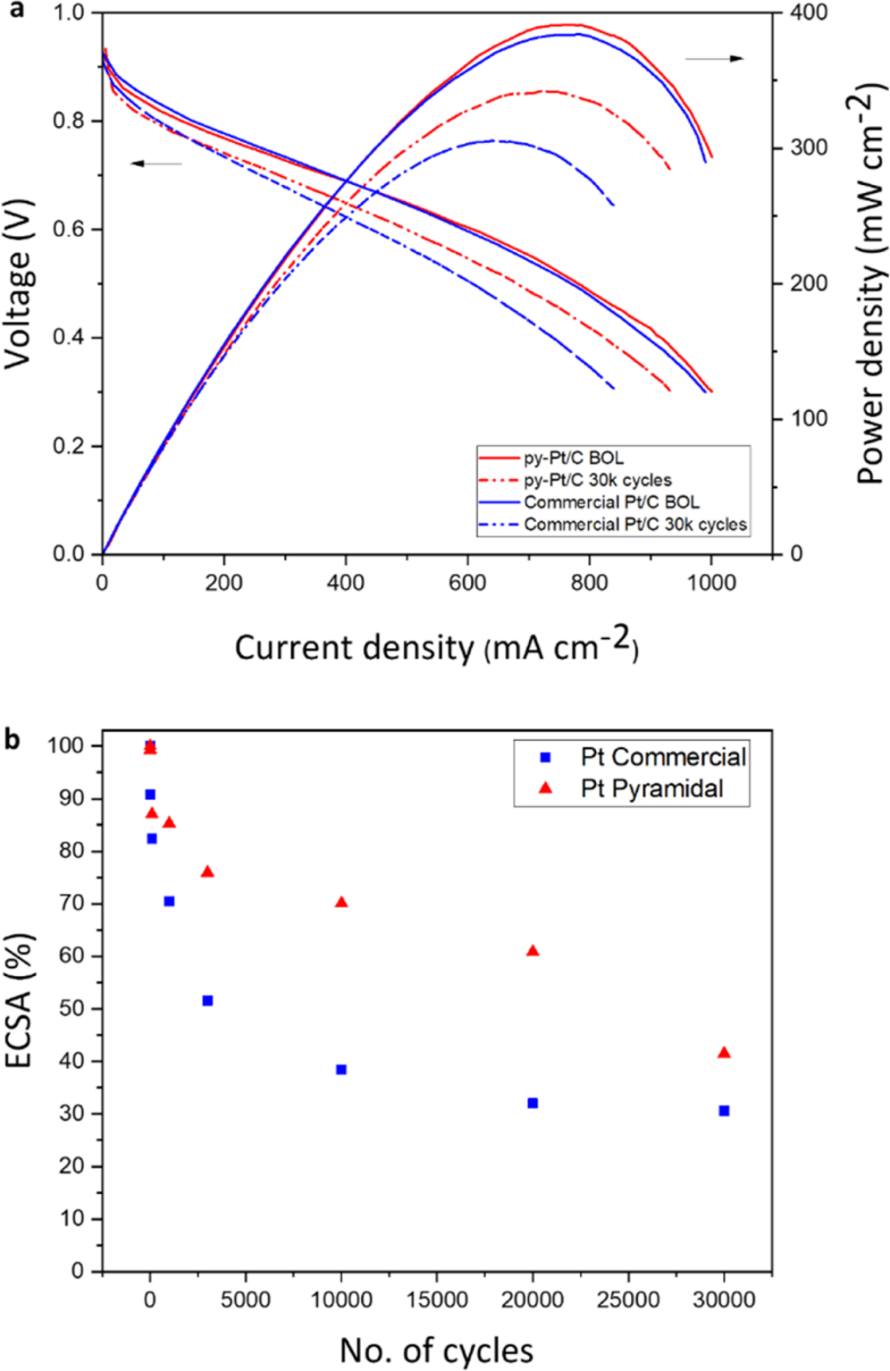
(a) Electrocatalytic fuel cell characterization. Full PEMFC polarization curves at the BOL and after 30k AST cycles for pyramidal (py-Pt/C) (in red) and commercial (Pt/C) (in blue) catalysts. Tests were performed using 100% relative humidity H_2_/air at a cell temperature of 80 °C with no back pressure. (b) ECSA values for the two different catalysts (Pt commercial in blue and Pt pyramidal in red) during ASTs shown as a percentage change (%).

Although the comparison with sp-Pt/C shows that py-Pt/C can offer increased durability, it should be noted that, unlike the commercial particles, the process for the supporting of the pyramidal Pt onto the carbon support is an in-house developed technique and therefore requires further optimization. As the method and quality of this catalyst supporting process can impact overall stability, it is important to compare py-Pt/C against commercial standards as they will be optimized for maximum lifetime and performance. Here, a commercial Pt/C catalyst with equivalent loading was formed into an MEA using the same procedure as for py-Pt/C; a comparison of the polarization curves for these two catalysts is shown in Figure 5a. At the BOL, it can be seen that the catalysts behave in an almost identical manner when compared using all metrics: peak power (391 mW cm^–2^ py-PT/C, 384 mW cm^–2^ commercial), peak current density at 0.3 V (1001 mA cm^–2^ py-PT/C, 990 mA cm^–2^ commercial), current density at 0.8 V (126 mA cm^–2^ py-PT/C, 141 mA cm^–2^ commercial), and voltage at 800 mA cm^–2^ (483 mV py-Pt/C, 478 mV commercial). This demonstrates that py-Pt/C is a high-activity catalyst. However, by 30 000 cycles, the performance of py-Pt/C is significantly better than the commercial catalyst, which has much more significant losses in all areas. The polarization curve at 800 mA cm^–2^ only decreases by 63 mV for py-Pt/C, compared to 131 mV for the commercial catalyst, and over the same number of cycles, the commercial Pt/C lost 21% of its power density, compared to just 12% for py-Pt/C (306 mW cm^–2^ vs 344 mW cm^–2^). As the MEAs were otherwise identical, these data demonstrate that the pyramidal Pt particles show significantly increased resistance to degradation.

The dramatically increased stability and degradation resistance of py-Pt/C can, in part, be explained by the relative change in ECSA vs the commercial equivalent (Figure 5b). Although the commercial Pt/C begins with a higher ECSA (66 m^2^ g^–1^ vs 41 m^2^ g^–1^ at the BOL), this decreases sharply over the first 3000 cycles, consistent with the other spherical catalyst tested (sp-Pt/C, Figure 4c,d), meaning that the commercial catalyst lost 62% of its surface by 10 000 AST cycles, compared to 29% for py-Pt/C (Figure 5b). The US DoE target for ECSA loss is <40% of the initial area. The commercial Pt/C fails this target at ∼3000 cycles, whereas py-Pt/C only breaches this threshold after ∼20 000 cycles.

Finally, the Py-Pt/C catalyst was assessed against a commercially prepared and optimized gas diffusion electrode (GDE) at a low catalyst loading (0.1 mg_Pt_ cm^–2^) and under enhanced accelerated stress test conditions (see the Materials and Methods section) designed to drive catalyst and support degradation.^47^ Importantly, these parameters were selected to be more representative of those in operating industry-relevant fuel cell applications; hence, a 25 cm^2^ cell was also utilized to demonstrate scalability. From the polarization curves shown in Figure S20, it can be seen that the performance of Py-Pt/C is close to that of commercial GDE, with only marginally lower current and power densities, despite the challenging low Pt loading conditions. More importantly, when assessed over the 30 000 enhanced AST cycles, using air at the cathode, the voltage losses and decreases in ECSA show the same trend as found with the DoE protocol; Py-Pt/C shows a lower voltage decrease (Figure S20a,b: 24 mV at 0.8 A cm^–2^ between BoL and 30 000 cycles vs 41 mV for the commercial GDE) and less ECSA loss with a more linear trend over time (Figure S20c–e). Moreover, when the performance of the same materials was assessed under lower gas humidification conditions (Figure S21), the Py-Pt/C catalyst appears to offer enhanced performance at lower (60%) relative humidities, with fewer losses in the mass transport and Ohmic regions. This may indicate an enhanced ability to maintain membrane humidification.

The above data, along with the results from the in situ TEM study (Figures S15–S19), demonstrates that the pyramidal Pt with a high percentage of dissolution and rearrangement-resistant {111} facets hinders catalyst agglomeration or corrosion, promoting overall full cell stability. Further optimization of the py-Pt/C catalyst, both in terms of its carbon supporting and loading, could reduce Pt migration further and hence further improve cell performance and lifetime.

## Conclusions

In this work, it has been demonstrated that single-crystal Pt nanoparticles with a high fraction of {111} facets and a size distribution close to that deemed ideal for application in PEMFCs can be synthesized via a one-pot, aqueous phase process without the use of polymers, surfactants, and any other difficult-to-remove agents. The process is highly scalable as no organic solvents, significant pollutants, high temperatures, or complex equipment are needed. Their yield is also high, the work-up is simple, and synthetic duration is low, meaning that there should therefore be a significant cost reduction with respect to syntheses that involve several steps and expensive reagents. This has been achieved by harnessing the shape-directing ability of sodium citrate together with a limited oxidative etching at relatively low temperatures.

New catalysts are rarely tested in full fuel cells, rather they are analyzed using methods such as rotating disk electrochemistry. Although these tests can be indicative of in situ performance, many catalysts that show excellent ex situ performance quickly fail when tested at the full cell level. Here, we have demonstrated that 3.4 nm pyramidal Pt nanoparticles with a high percentage of {111} domains can provide current and power densities that match, if not exceed, those provided by MEAs constructed using optimized commercial Pt/C catalysts in full single-cell PEMFCs. More importantly, these new catalysts are shown to offer significantly enhanced durability across 30 000 AST cycles, even when compared to commercial catalysts and commercially prepared catalyst systems. It has been previously shown that the Pt(111) surface is resistant to corrosion^37,38^ and this is suggested as the mechanism driving the increase in MEA stability. With further catalyst/support optimization to minimize catalyst coalescence, this method of catalyst preparation holds great promise for widespread application, driving the move to “green” fuel cell catalysts.

## Materials and Methods

### Materials

Chloroplatinic acid hexahydrate, BioXtra product line (with strict specifications about the absence of major contaminants and the presence of negligible traces of other metals), sodium citrate tribasic dehydrate BioUltra, sodium borohydride, and citric acid anhydrous were purchased from Merck/Sigma-Aldrich and used as received. Vulcan carbon black XC72R was purchased from FuelCellStore. Deionized (DI) water with a resistivity of 18.2 MΩ·cm was used throughout the experiment.

### Pyramidal Pt NP Synthetic Procedure

The 3 nm pyramidal Pt NPs were synthesized by adding 55 μL of hexachloroplatinic acid aqueous solution (0.5 M, BioXtra grade, Sigma-Aldrich) to 90 mL of Milli-Q water at 90 °C. After 60 s, an aqueous solution (2 mL) at a concentration of 35 mM sodium citrate and 3 mM citric acid was added, immediately followed by quick addition of 1.1 mL of a 22 mM aqueous solution of NaBH_4_, freshly dissolved. The vessel was then capped, and the reaction was left to proceed without further addition for 10 min. After this time, the solution was removed from the glycerol bath and cooled to room temperature. After cooling, the NPs were extensively washed using 30K Amicon Ultra Centrifugal Filters and a 2 mM sodium citrate solution and then stored at 4 °C.

### Deposition of Pyramidal Pt NPs on Carbon Black

The water dispersion of Vulcan carbon black XC72R was achieved by adding the material to ultrapure water, followed by alternate stirring and sonication for 2 h. The pyramidal Pt NPs were then added to the carbon black aqueous suspension with a targeted loading of 20% w/w (Pt/C), and this mix was sonicated for a further 15 min. Then, NaOH pellets (in a quantity sufficient to obtain a final concentration of 1 M) were added to the glass vial to remove the citrate coating from the nanoparticle surface to favor the deposition of the nanoparticles on the carbon support, and the mixture was left overnight to deposit and sediment. After this time, the composite material was completely separated from the aqueous solution at the bottom of the vial. This precipitate was then washed five times with ultrapure water. TGA measurements were performed to calculate the loading of Pt onto the carbon support (see the Supporting Information).

### Transmission Electron Microscopy (TEM) Analysis of Nanoparticle Size and Surface Structure

Bright-field TEM (BF-TEM) analysis was carried out using a JEOL JEM 1011 microscope with a W filament, operating at 100 kV. High-angle annular dark-field scanning TEM (HAADF-STEM) and high-resolution TEM (HR-TEM) imaging analyses were carried out using an image-Cs-corrected JEOL JEM-2200FS microscope with a Schottky emitter and operating at 200 kV. The lateral size of the nanocrystals was obtained by manually applying a threshold on the HAADF-STEM images, followed by automatic measurements using ImageJ.^64^ From the VESTA^49^ schematic model of pyramidal NP with an edge length equal to 3.4 nm, we calculate that the total number of atoms is equal to 1079, while the number of atoms on the surface is 474. This means that almost 50% of the atoms are located on the surface of the nanomaterial.

### Electrochemical Characterization of {111} Pt Surface Facets

The electrochemical measurements to determine the percentage of {111} Pt surface facets were performed using an Autolab potentiostat (PGSTAT302N, Metrohm Autolab). The experiments were carried out at room temperature with a conventional three-electrode electrochemical cell. Potentials were measured against a reversible hydrogen electrode (RHE) connected to the cell through a Luggin capillary. A platinum wire was employed as a counter electrode. Before each experiment, the glassy carbon working electrode was mechanically polished to a mirror finish with 1.0 and 0.3 μm alumina slurries (Buehler) and sonicated in Milli-Q water (Millipore) to remove all polishing residues. It was then prepared by depositing 4 μL of the Pt nanoparticle suspension that was dried under an Ar atmosphere at room temperature until the solvent had completely evaporated. The voltammetric characterization of the samples was performed in 0.5 M H_2_SO_4_. All electrolyte solutions were prepared using Milli-Q water and Merck p.a. sulfuric acid, deaerated with Ar (Air Liquide N50) before being used.

To demonstrate the surface cleanliness of the Pt samples, CO stripping experiments were performed. In brief, CO (Air Liquide N47) was bubbled in the electrolyte (0.5 M H_2_SO_4_) at 0.05 V for 1–2 min. Once the complete CO blockage of the surface was verified by cycling the electrode between 0.05 and 0.35 V, CO was removed from the solution by bubbling Ar for at least 15–20 min. Finally, adsorbed CO was electrochemically oxidized in a single sweep up to 0.9 V at 20 mV s^–1^ (Figure S10).^65^

The electroactive surface area of the Pt nanoparticles was determined by measuring the charge involved in the so-called hydrogen UPD region and assuming 230 μC cm^–2^ for the total charge after the subtraction of the double layer contribution as described in the previous contributions.^59^

The quantification of the {111} surface sites on the surface of the Pt nanoparticles was carried out by employing the irreversible adsorption of bismuth (Bi). Bi adsorption was achieved by contacting the electrode coated with the Pt nanoparticles with a Bi_2_O_3_ saturated solution (0.5 M H_2_SO_4_) at the open-circuit potential. The maximum Bi coverage was reached with immersion times of about 2–4 min. The electrode was then thoroughly rinsed with ultrapure water before being immersed in the electrochemical cell containing a 0.5 M H_2_SO_4_ solution and cycled between 0.05 and 0.75 V at 50 mV s^–1^.^57^

### Rotating Disk Accelerated Stress Test Analysis

Electrochemical rotating disk measurements were carried out using a Gamry potentiostat (Interface1000) coupled with a rotating disk electrode (RDE, Pine, AFMSRCE 3005) in a three-electrode cell setup. Two milligrams of each catalyst was dispersed in 1 mL of 0.05 wt % Nafion in a 1:1 water/IPA mixture to form a homogeneous catalyst ink by sonicating for 40 min. Then, 7.4 μL were drop-cast onto a polished (Al_2_O_3_ micro-polish, Bueler) 0.1963 cm^2^ glassy carbon working electrode, achieving a loading of 15 μg_Pt_ cm^–2^ for both catalysts. This was used in a three-electrode cell containing a 0.1 M HClO_4_ electrolyte vs a reversible hydrogen electrode (RHE, Gaskatel) and a Pt mesh counter electrode.

Working electrodes were first electrochemically activated via rapid cycling (500 mV s^–1^, 50 cycles) between 0.05 and 1.2 V_RHE_. Cyclic voltammograms were obtained by cycling the working electrode between 0.025 and 1.2 V_RHE_ at room temperature, under N_2_ flow, at a scan rate of 20 mV s^–1^. To investigate ORR activity, linear sweep voltammetry (LSV) was performed at room temperature under constant O_2_ flow at 1600 rpm. The scans were performed at 10 mV s^–1^ between 0.1 and 1.1 V_RHE_. To examine the durability of the two catalysts, corrosion experiments were carried out between 0.6 and 1 V_RHE_. Here, the working electrode was cycled at 50 mV s^–1^, and then at intervals, a full CV was taken between 0.025 and 1.2 V_RHE_ at 20 mV s^–1^, from which the hydrogen adsorption peaks (0.075–0.4 V_RHE_) were used to calculate the ECSAs. A total of 30 000 cycles were carried out for each durability test, following the DoE protocol.^48^

TEM imaging was performed to evaluate the effect of the accelerated stress test on the catalysts. Once a homogeneous ink was prepared for both catalysts, they were drop-cast onto a holey carbon grid (Agar Scientific). For TEM imaging, gold mesh “finder” grids were used to allow the same area of the grid to be assessed before and after cycling. Images were collected before and after 30 000 0.6–1 V_RHE_ potential cycles, where the TEM grid was directly used as the working electrode.^66^ TEM images were acquired using a Jeol JEM 2100 TEM equipped with a LaB6 source.

### In Situ Fuel Cell Testing

All in-house prepared cathode GDEs used the same ionomer-to-carbon ratio (I/C = 0.8) in the catalyst layer (CL). A commercial platinum/carbon catalyst (Johnson Matthey, 20 wt % Pt/C HiSpec 3000) was used to provide a baseline comparison for the carbon-supported spherical and pyramidal Pt nanoparticle catalysts. The three different inks were prepared using the same amount of catalyst powder (commercial, spherical, or pyramidal Pt supported on carbon) mixed with an aqueous Nafion solution (5 wt %, Fuel Cell Store); DI water and IPA (1:1 ratio) were used as the solvent. This catalyst ink was homogenized via sonication for 60 min. Freudenberg H23C2 (250 μm) was used as a gas diffusion media, consisting of a carbon-fiber paper, PTFE-treated, with a carbon microporous layer on one side. Catalyst coating was undertaken using a robotic spraying system (Sono-Tek “Exacta-coat,” Sono-Tek), equipped with an AccuMist ultrasonic nozzle. All in-house prepared gas diffusion electrodes GDEs had a loading of 0.4 mg_Pt_ cm^–2^.

For the 5 cm^2^ cell experiments, HyPlat (HyPlat, South Africa) GDEs, which contain the gas diffusion layer (GDL), microporous layer (MPL), and a CL with a Pt loading of 0.4 mg_Pt_ cm^–2^, were cut to size (cm^2^) and used as the anode. A GORE-SELECT membrane (Gore), 20 μm thick, with an area of 16 cm^2^ was used. For the 25 cm^2^ cell experiments, HyPlat GDEs with a Pt loading of 0.2 mg_Pt_ cm^–2^ were cut to an area of 25 cm^2^ and used as the anode electrode and a GORE-SELECT membrane, 20 μm thick, with an area of 36 cm^2^ was used. Here, either a HyPlat GDE with 0.1 mg_Pt_ cm^–2^ loading or an in-house prepared pyramidal Pt GDE with the same loading was used at the cathode.

In all cases, the MEA components were hot-pressed for 3 min at 130 °C and 400 psi for all experiments. The MEAs with 5 cm^2^ active area were assembled in single-cell hardware with a single-channel serpentine flow field using 4 N m torque (5 cm^2^ fuel cell fixtures supplied by Scribner), whereas the larger 25 cm^2^ cell used a triple-channel serpentine flow field and 25 cm^2^ fuel cell fixtures supplied by Scribner. The pitch was kept around 25% of the total GDE thickness using Teflon gaskets. The assembled cell was then connected to a Scribner Associates 850e (Scribner) test station, with an in-built potentiostat (Scribner 885). All MEAs were conditioned in an identical way by holding at a constant voltage of 0.6 V for 1 h until a stable behavior was reached, and the increasing current density was monitored. Three polarization curves were measured, and the MEA was considered to be broken if there was a deviation of <5 mV, in accordance with the US DoE testing recommendation.^48^

For the 5 cm^2^ cell (i.e., tests with the in-house prepared GDEs, Py-Pt/C, Sp-Pt/C, Pt/C), the temperature was set at 80 °C, and the anode and cathode were fed with humidified (100% relative humidity) H_2_ and air, with a stoichiometry of 1.5 and 3, respectively. The outlet of both the anode and cathode was at atmospheric pressure. Accelerated stress tests (ASTs) were run following the US DoE testing metrics.^48^ Polarization curves and cyclic voltammetry curves were used for electrochemical characterization and calculating the electrochemical surface area (ECSA) via the UPD method. Polarization measurements on the different MEAs were performed between the open-circuit voltage (OCV) and 0.3 V at intervals of 0.025 V and 30 s hold at each voltage interval point. For the ECSA measurements, nitrogen was then flowed over the cathode at a fixed flow rate of 0.2 L min^–1^ and was fully purged of all oxygen until the OCV was <0.15 V. Cyclic voltammetry was then carried out by sweeping the voltage between 0.05 and 1 V at a scan rate of 20 mV s^–1^. In the 25 cm^2^ cell, enhanced ASTs were run in H_2_ and air for 0.1 mg_Pt_ cm^–2^ commercial GDE and Py-Pt/C, following the protocol published by Zhang et al. and assessed by the National Renewable Energy Laboratory (NREL), Argonne National Laboratory (ANL), and Los Alamos National Laboratory (LANL).^47^ The cell temperature was set at 80 °C, H_2_ and air were used as the anode and cathode gases, respectively, and the cell was tested at 100% relative humidity. A 100 kPa total pressure was applied. Square-wave cycles were performed in air using steps between 0.60 V (3 s) and OCV (3 s) and a rise time of 0.5 s or less. Fuel cell polarization curves and cathode cyclic voltammetry curves in N_2_ are reported at specific time intervals. A total of 30 000 cycles were performed. The performance of these cells was also assessed at 100, 80, 70, and 60% RH.

## Supporting Information Available

Supplementary TEM images, synthetic protocols, XRD analysis, CO stripping voltammetric profile, TEM images of pyramidal Pt NPs on amorphous carbon, thermogravimetric analysis, linear sweep voltammograms, cyclic voltammograms, in situ TEM analysis for Pt catalysts collected at BOL and after 30 000 cycles, full PEMFC polarization curves at the BOL and after 30k H_2_/air AST, cyclic voltammograms measured during the enhanced accelerated stress test and associated ECSA percentage change, and polarization curves at different relative humidity levels (PDF)
