# Supplementary material for: Ultrasmall, Coating-Free, Pyramidal Platinum Nanoparticles for High Stability Fuel Cell Oxygen Reduction
Source: ACS Appl Mater Interfaces. 2022 Aug 3;14(32):36570–81. doi: 10.1021/acsami.2c07738 (PMC9975930; doi:10.1021/acsami.2c07738)
Supplement: Supplementary file 1 — am2c07738_si_001.pdf [file am2c07738_si_001.pdf]

SUPPORTING INFORMATION

Ultra-small, coating-free, pyramidal platinum nanoparticles for high stability fuel cell oxygen reduction

Valentina Mastronardi[1,2,⊥], Emanuele Magliocca[3,⊥], José Solla Gullon[4], Rosaria Brescia[5], Pier Paolo Pompa[1], Thomas S. Miller[3,†,*], Mauro Moglianetti[1,•,†,*]

1. Istituto Italiano di Tecnologia, Nanobiointeractions&Nanodiagnostics, Via Morego 30 – 16163 Genova, Italy

2. Department of Chemistry and Industrial Chemistry, University of Genova, Via Dodecaneso 31, 16146 Genova, Italy

3. Electrochemical Innovation Laboratory, Department of Chemical Engineering, University College London, Torrington Place, WC1E 7JE London, UK

4. Institute of Electrochemistry, University of Alicante, Apdo. 99, E-03080 Alicante, Spain

5. Electron Microscopy Facility, Istituto Italiano di Tecnologia, Via Morego 30, 16163 Genova, Italy

[⊥] Equally Contributing Authors

[•] Current address: HiQ-Nano srl, Via Barsanti, 1, Arnesano, Lecce, Italy

[†] These authors jointly supervised this work.

* mauro.moglianetti@hiqnano.com, t.miller@ucl.ac.uk

Table S1. Comparison of RDE and fuel cells (membrane electrode assembly, MEA) performance results of pyramidal Pt/C (this work) with shaped Pt/C catalysts from the literature.

**Parameter**	**RDE** $\mathbf{j_{mass}}$	**RDE** $\mathbf{j_{mass}}$ **after 5-30k CV Retention (%)**	**RDE ECSA**	**RDE ECSA after 5-30k CV Retention (%)**	**RDE** $\mathbf{j_{spec}}$	**RDE** $\mathbf{j_{spec}}$ **after 5-30k CV Retention (%)**	**Full cell Membrane Electrode Assembly Power density Peak**	
Unit	A mg_{Pt}^{-1}	A mg_{Pt}^{-1} %	$m^2\ g_{Pt}^{-1}$	$m^2\ g_{Pt}^{-1}$ %	mA cm^{-2}	mA cm^{-2}	mW cm^{-2}	
20% pyramidal Pt/C 3.4 nm edge size	1.25	0.95 76%	42.3	36.6 86.5%	3.20	2.50 78%	411	This work
13 wt% octahedral Pt/C 5.2 nm diameter	0.36 ± 0.09	0.31 ± 0.04 86%	36 ± 2	32 ± 3 89%	1.10 ± 0.26	0.94 ± 0.07 85%	Non-tested	[1]
50 wt% spherical Pt/C (JM) 4.4 nm diameter	0.31 ± 0.01	0.27 ± 0.01 87%	67.7 ± 6.9	67.3 ± 9.0 99%	0.46 ± 0.06	0.40 ± 0.07 87%	Non-tested	[2]
46.7 wt% spherical Pt/C 6.4 nm diameter	0.04 ± 0.01	-	17 ± 2	-	0.24 ± 0.02	-	Non-tested	[3]

15 wt% octahedral Pt/C sub-10 nm diameter	0.25	-	16.80	-	1.49	-	Non-tested	[4]
15 wt% octahedral-Pt/C 30.8 nm edge length	0.30	-	16.87	-	1.66	-	Non-tested	[5]
Icosahedral Pt nanocages 13.8 nm edge length	1.28	0.76<br>59%	36.5	29.7<br>82%	3.50		Non-tested	[6, 7]
Platinum porous nanosheets	2.07	87.2%	70.2	56.5<br>80.5%	3.1	90.2%	Non-tested	[7, 8]
Platinum nanoplates	1.62	1.17	30.6	83%	5.3		Non-tested	[7, 9]

XRD characterization of ultra-small pyramidal Pt NPs

X-ray diffraction (XRD) patterns were recorded on a Malvern-PANalytical 3rd generation Empyrean X-ray diffractometer, equipped with a 1.8kW CuKα ceramic X-ray tube, PIXcel^{3D} detector and operating at 45 kV and 40 mA. The diffraction patterns were collected in air at room temperature using Bragg Brentano (BB) geometry. Sample was prepared by drop casting the concentrated NC solution onto a zero-diffraction silicon substrate followed by drying under slightly reduced pressure. XRD data analysis was carried out using HighScore 5.1 software from PANalytical. An average crystallite domain size of 2.7nm was estimated using the Scherrer equation.

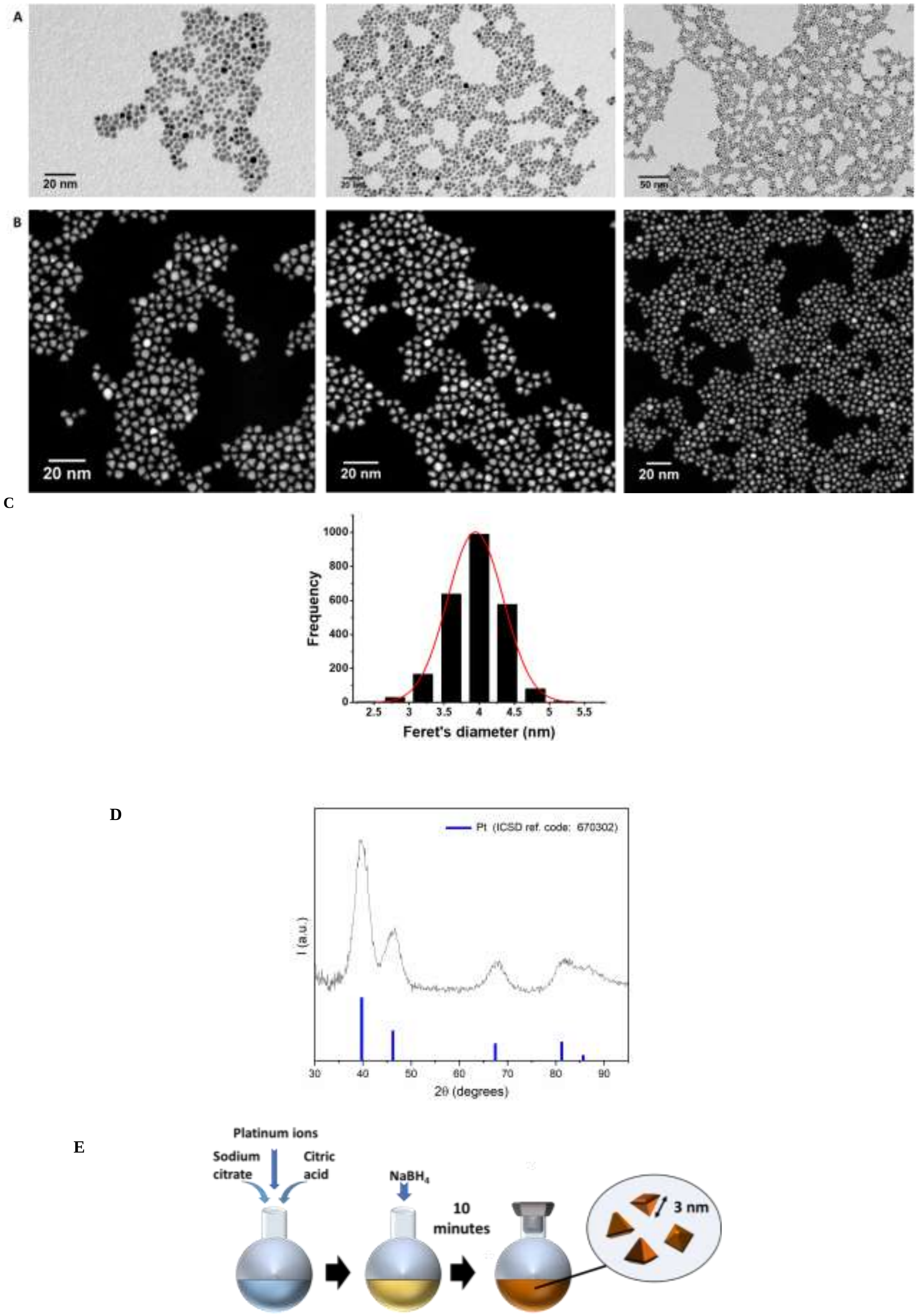
A
20 nm
50 nm
B
20 nm
20 nm
20 nm
C
Frequency
Feret's diameter (nm)
D
Pt (ICSD ref. code: 670302)
I (a.u.)
2θ (degrees)
E
Platinum ions
Sodium citrate
Citric acid
NaBH4
10 minutes
3 nm

Figure S1. A) Bright-field transmission electron microscopy (BF-TEM) and B) High-angle annular dark-field scanning transmission electron microscopy (HAADF STEM) images of **ultra-small pyramidal Pt NPs** and C) relative size distribution of Feret's diameter. D) XRD diffraction pattern obtained from Pt pyramidal nanoparticles drop-casted onto a zero-diffraction silicon substrate. An average crystallite domain size of 2.7nm was estimated using the Scherrer equation. E) Scheme of the process for the production of ultra-small pyramidal Pt NPs.

Table S2. Summary of the synthetic parameters and physico-chemical conditions playing a role in the anisotropic growth of pyramidal nanoparticles.

NPs shape	Oxygen concentration	T (glycerol bath)	Purity of Pt precursor	[Pt] uM	[$NaBH_4$] uM	[Na3C6 H5O7] uM	Time (min)	Fig
Octahedral monodisperse	Fixed (closed vessel)	90°C	Purified	300	526	76	10	1
Highly polydisperse	Variable (open vessel)	120°C	Purified	300	526	76	10	S2
Highly polydisperse	Variable (open vessel)	90°C	Purified	300	526	76	10	S3
Highly polydisperse	Fixed (closed vessel)	90°C	Purified	300	526	76	30	S4
Highly polydisperse	Fixed (closed vessel)	80°C	Purified	300	526	76	10	S5
Highly polydisperse	Fixed (closed vessel)	120°C	Purified	300	526	76	10	S5
Highly polydisperse	Fixed (closed vessel)	90°C	Presence of impurities	300	526	76	10	S6
Highly polydisperse	Fixed (closed vessel)	90°C	Purified	65	526	76	10	S7
Highly polydisperse	Fixed (closed vessel)	90°C	Purified	560	526	76	10	S7
Highly polydisperse	Fixed (closed vessel)	90°C	Purified	300	280	76	10	S8
Highly polydisperse	Fixed (closed vessel)	90°C	Purified	300	500	76	10	S8
Highly polydisperse	Fixed (closed vessel)	90°C	Purified	300	526	175	10	S9
Highly polydisperse	Fixed (closed vessel)	90°C	Purified	300	526	18	10	S9

Synthesis and characterization of quasi-spherical Pt nanoparticles

Method: The synthesis of the quasi-spherical Pt nanoparticles has been performed using a methodology previously published.[1] Pt spherical NPs were synthesized by adding 53 μL of hexachloroplatinic acid aqueous solution (0.5 M) (producer and dealer: Alfa Aesar) to 90 mL of Milli-Q water at boiling point (glycerol bath temperature set at 110°C). After 1 min, 2.2 mL of 35 mM sodium citrate and 3 mM citric acid aqueous solution was added, immediately followed by a quick addition of 1.1 mL of 22 mM aqueous solution of NaBH4, just dissolved. After 10 min, the solution was cooled to room temperature.

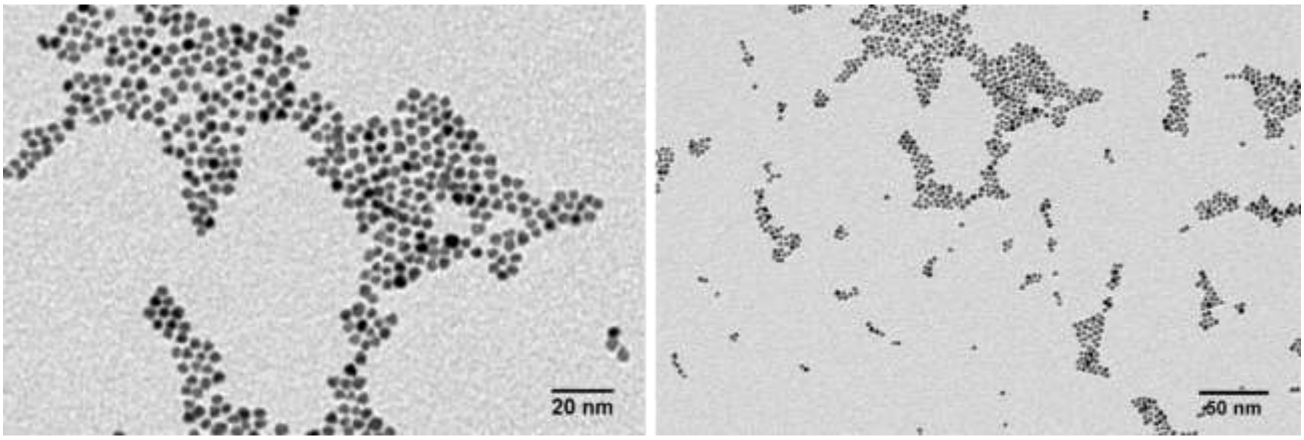

Figure S2. Representative TEM images of the quasi-spherical Pt nanoparticles with average diameter equal to 3.3 ± 0.4 nm.

Effect of oxygen during the reaction on the shape and geometry of the NPs

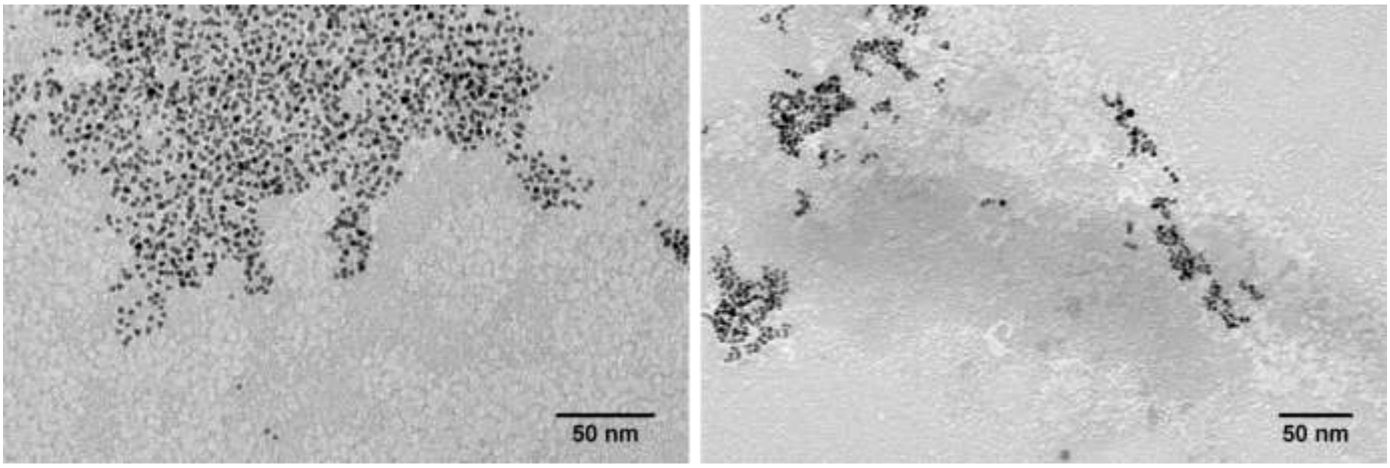

Figure S3. Representative TEM images of the quasi-spherical Pt nanoparticles with an open vessel setup.

Effect of reaction time on the shape and geometry of the NPs

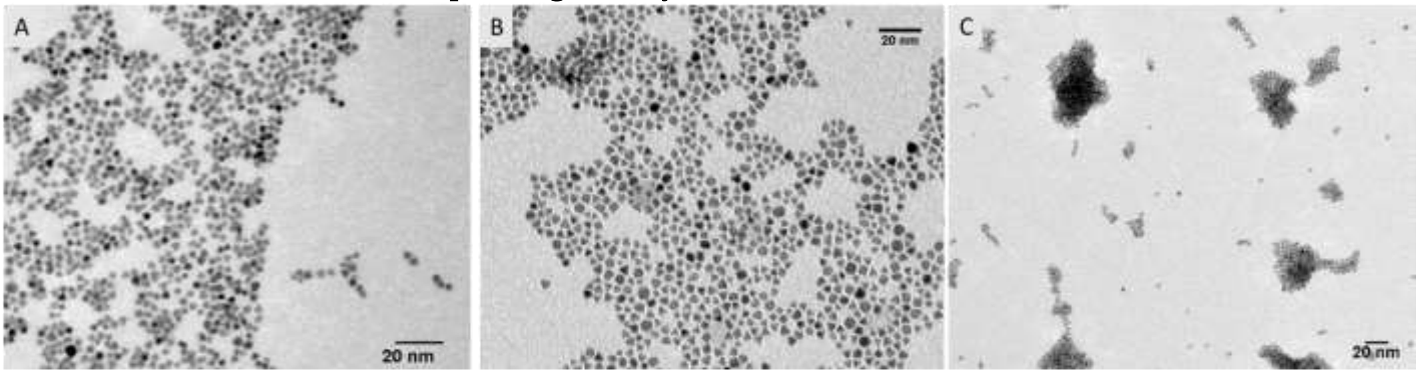

Figure S4: TEM images of Pt nanoparticles synthesized with reaction time equal to: A) 2 minutes (leading to nanoparticles with an average size of 3 ± 0.5 nm), B) 5 minutes (leading to nanoparticles with average size of 3.5± 0.6 nm), C) 30 minutes (leading to nanoparticles with an average size of 3.1 ± 0.5 nm).

Effect of the reaction temperature

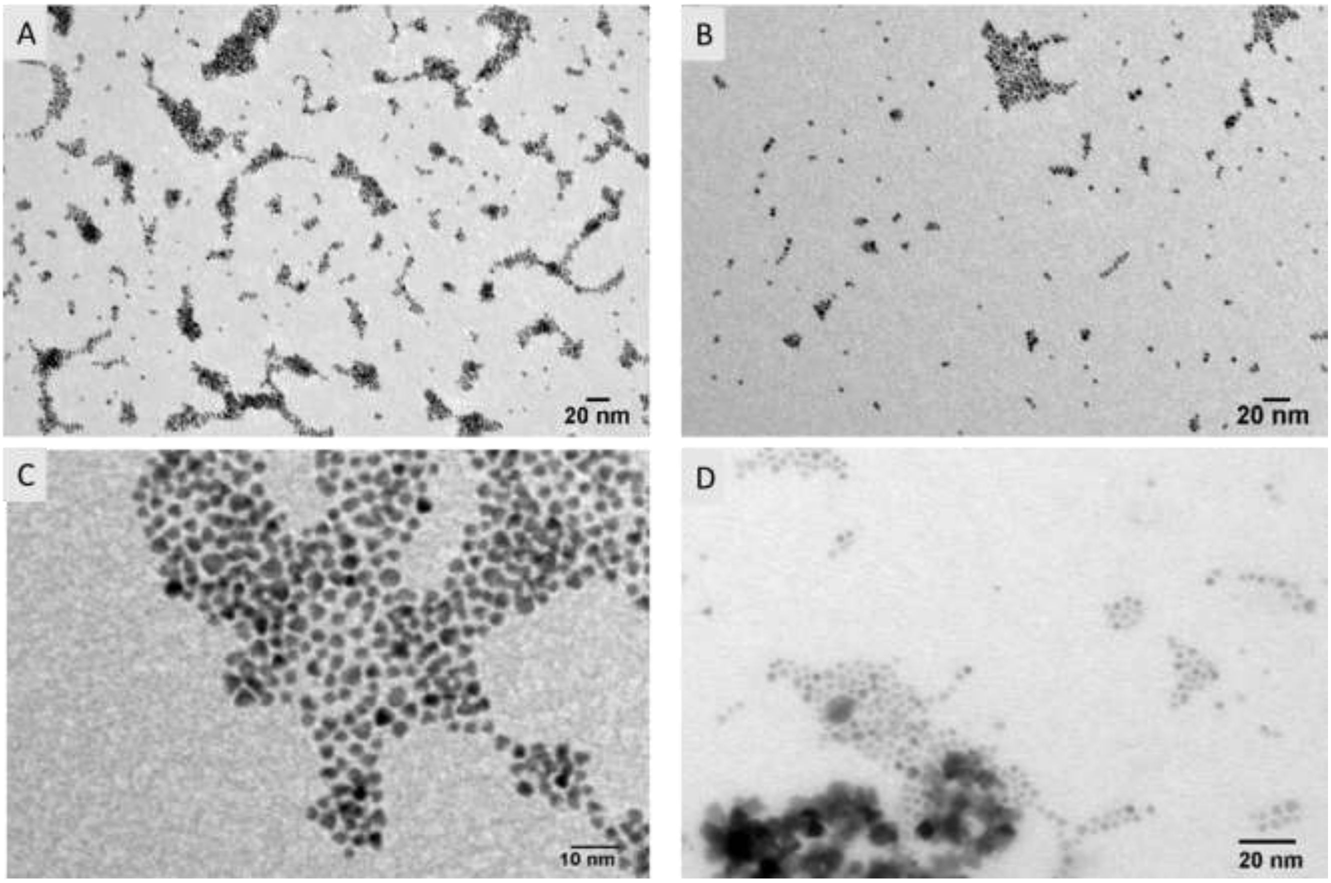

Figure S5: TEM image of Pt nanoparticles synthesized at the following temperatures: A) 60°C (leading to nanoparticles with average size of 3± 0.4 nm and with high polydispersity in shape), B) 80 °C (leading to nanoparticles with average size of 2.9 ± 0.7 nm), C) 100°C (leading to nanoparticles with average size of 2.7± 0.4 nm), D) 120°C set for the glycerol bath (leading to polydisperse and aggregated nanoparticles).

Effect of precursor impurities

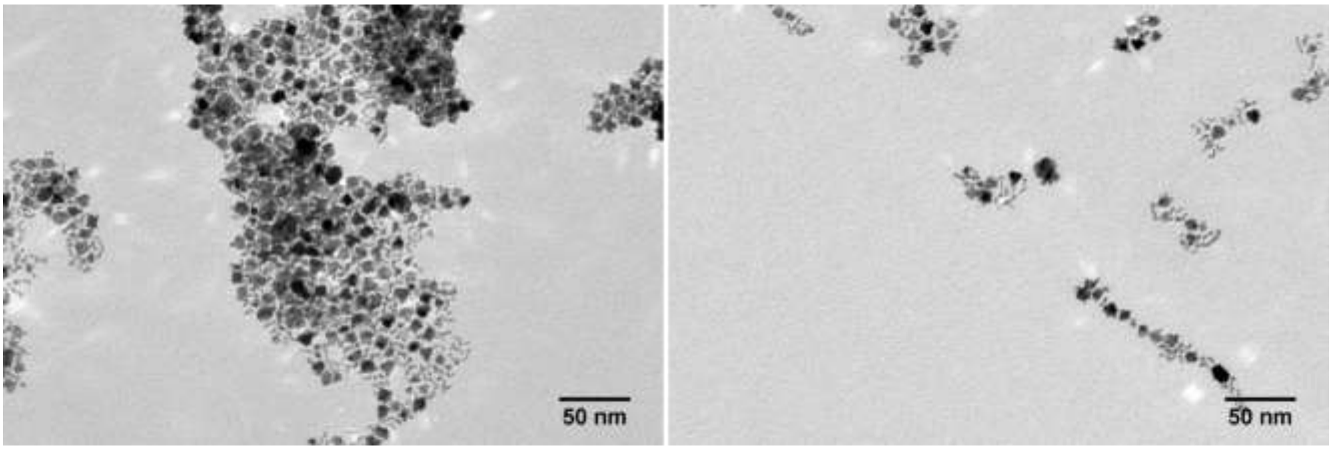

Figure S6: TEM image of Pt nanoparticles synthesized with a Pt precursor contaminated by impurities.

Effect of the concentration of the Pt precursor

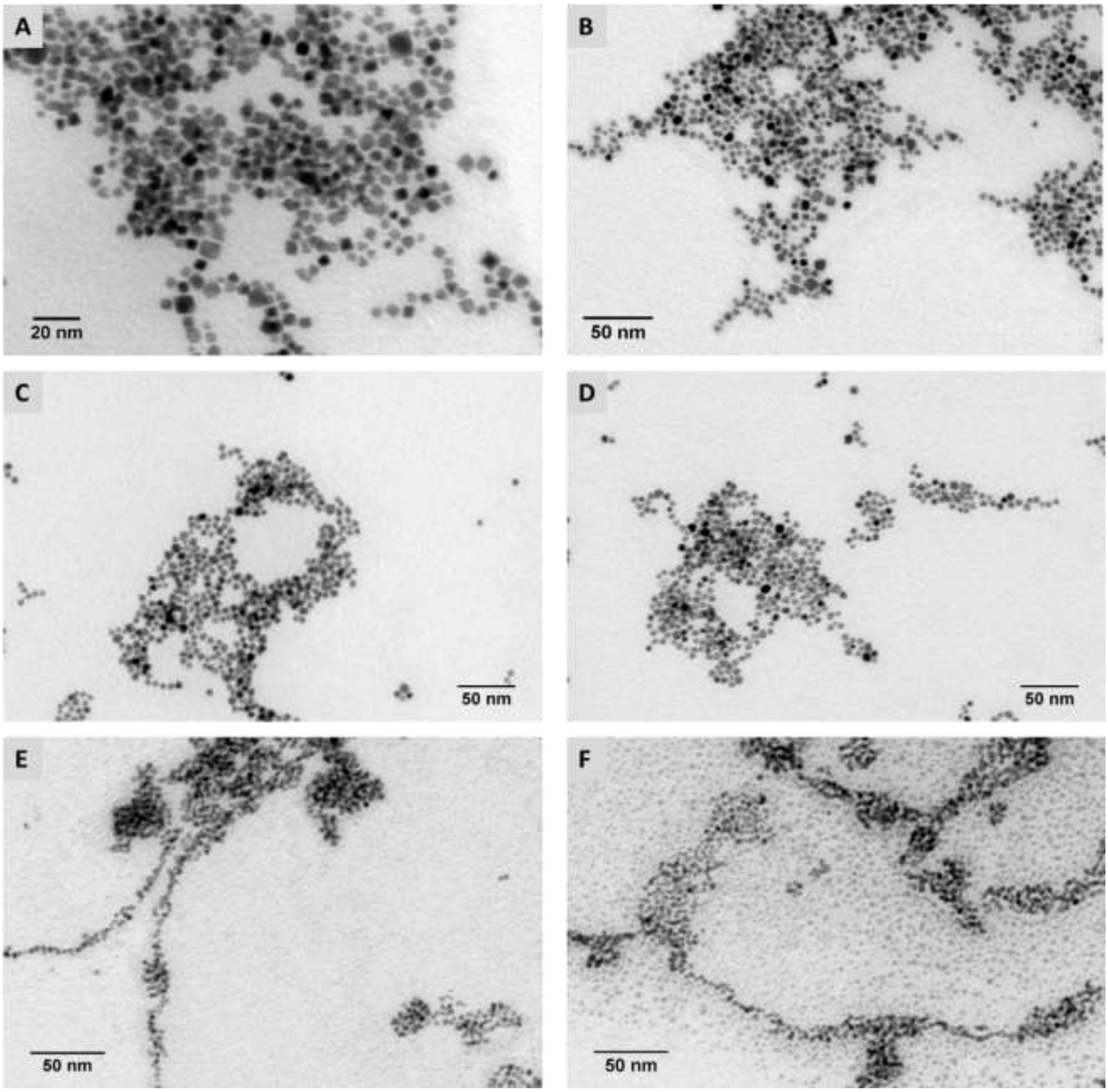

Figure S7: TEM image of Pt nanoparticles synthesized with different concentration of H_2PtCl_6: A-B) 65 µM (leading to two populations of NPs), C-D) 150 µM (leading to nanoparticles with an average size of 5.7± 1.7 nm), E-F) 560 µM (leading to unshaped and polydisperse Pt NPs).

Effect of the concentration of $NaBH_4$

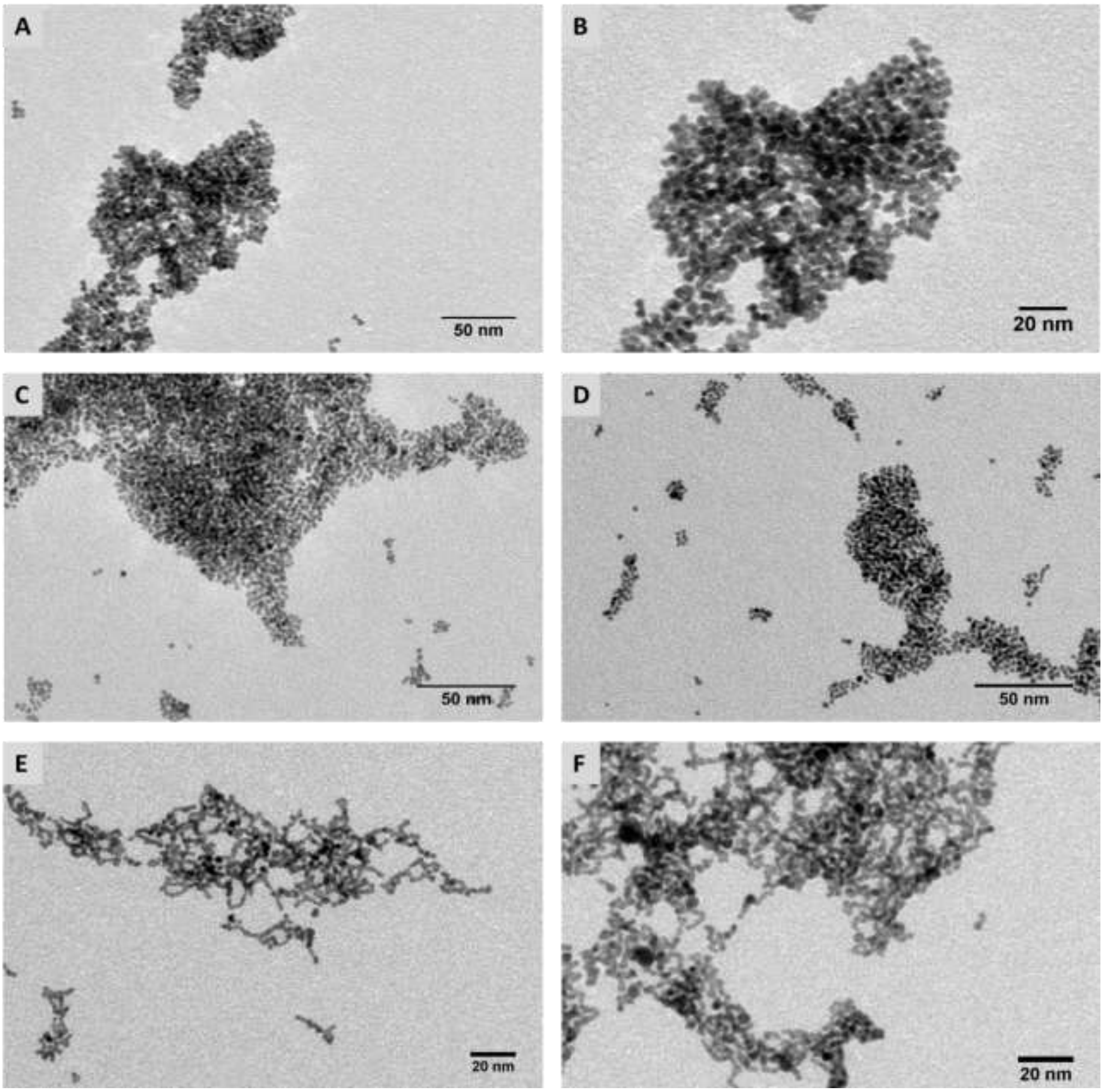

Figure S8: Bright-field TEM images of Pt nanoparticles prepared using the following concentration of $NaBH_4$: (A, B) 280 µM (leading to nanoparticles with average size of 4.1± 0.9 nm), (C, D) 500 µM (leading to aggregated nanoparticles), and (E, F) 1mM (leading to aggregated nanoparticles).

Effect of the sodium citrate concentration

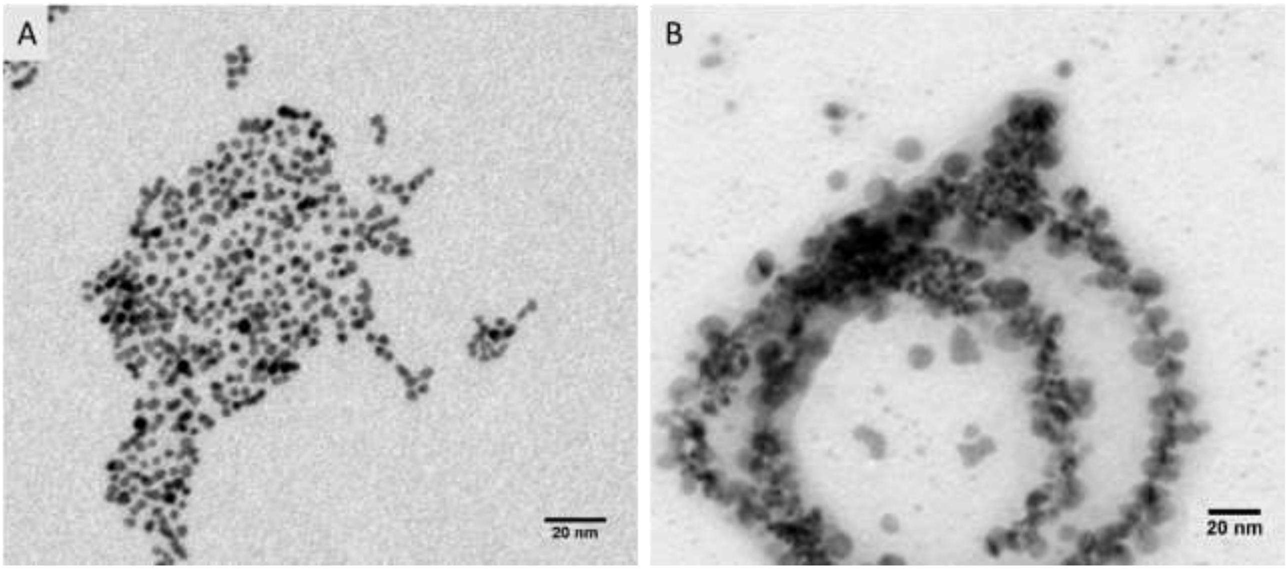

Figure S9: TEM image of Pt nanoparticles synthesized with the following concentration of Sodium Citrate: A) 175mM (leading to polydisperse in shape Pt NPs with average size of 2.9± 0.5 nm), B) 17.5 mM (leading to amorphous aggregates of Pt NPs).

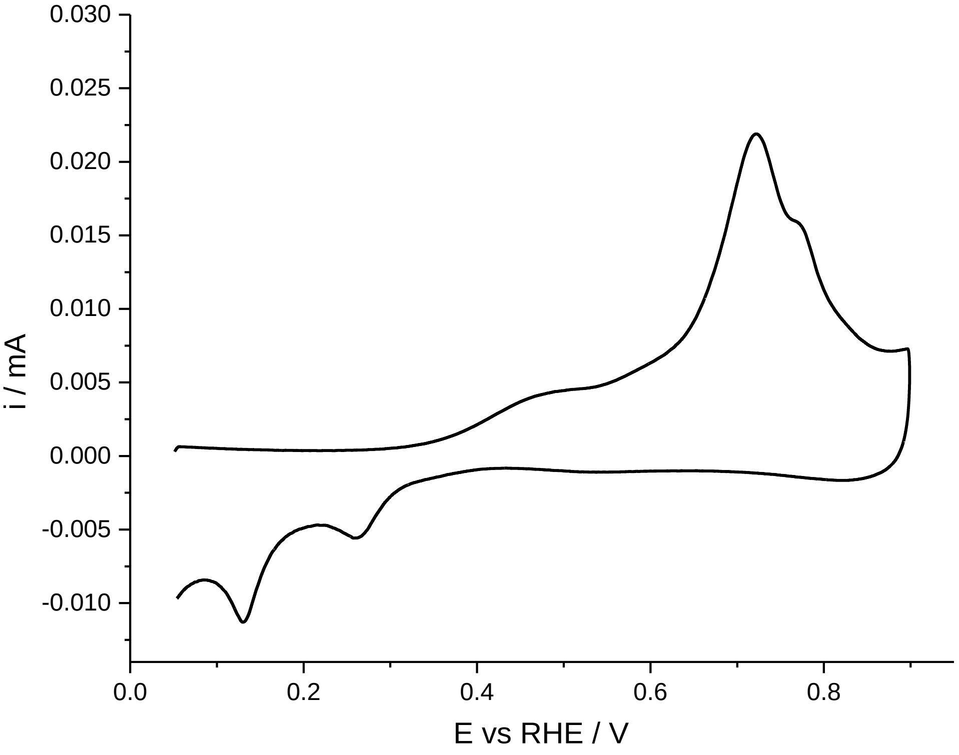

Figure S10: CO stripping voltammetric profile for the ultra-small pyramidal Pt NPs. Test solution: 0.5 M H_2SO_4, sweep rate 20 mV s^{-1}.

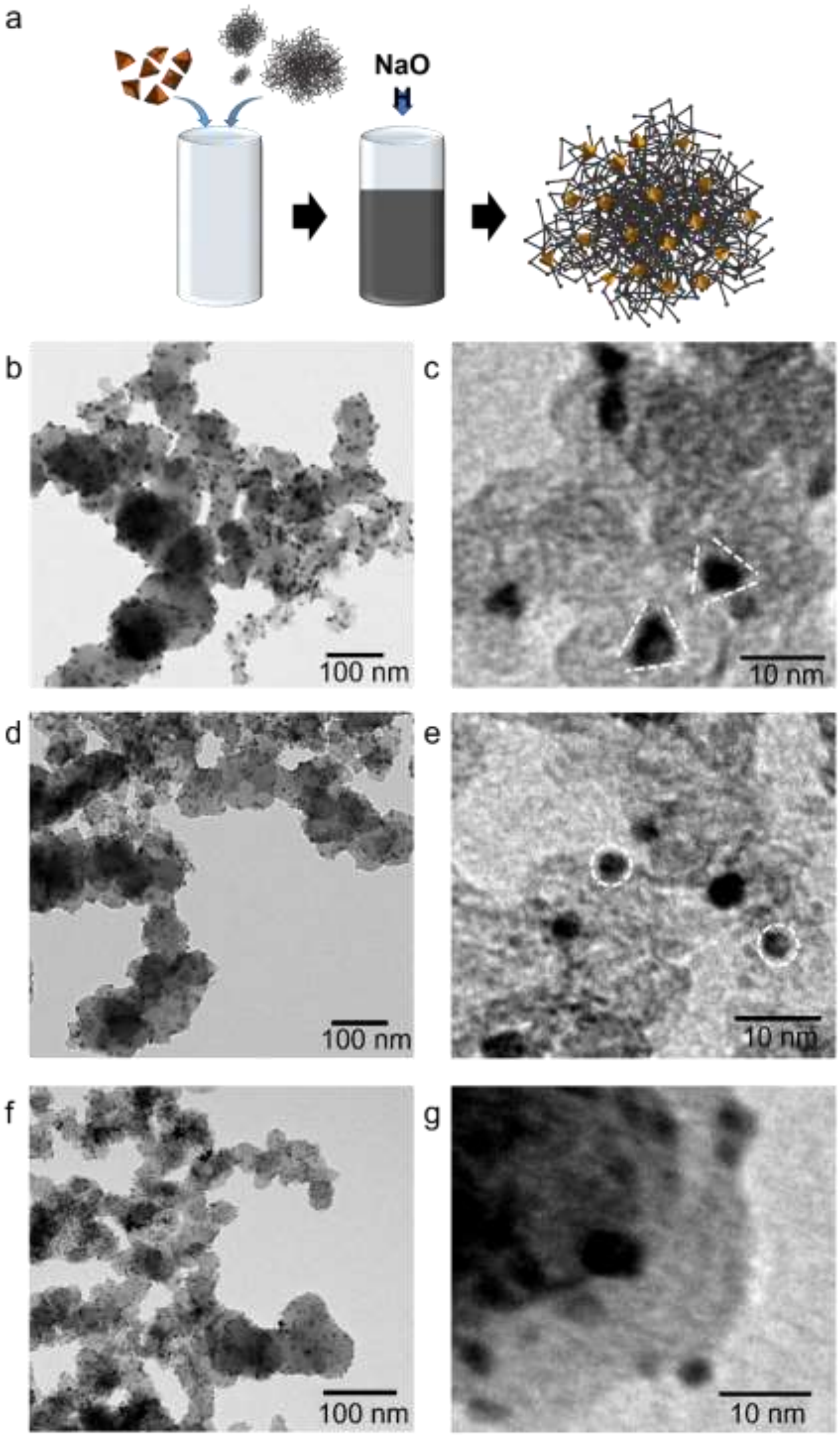

Figure S11. Deposition of pyramidal Pt NPs on amorphous Carbon support. (a) Schematic representation of the simple process for the deposition of pyramidal Pt NPs on amorphous carbon. (b) (c) Morphology of pyramidal Pt/C as visualized by BF-TEM at two different magnifications. The Bf-TEM image shows the pristine initial state of the pyramidal Pt NPs in black and the Vulcan support in gray. (d) (e) Morphology of spherical Pt/C as visualized by BF-TEM at two different magnifications. (f) (g) Morphology of commercial Pt/C as visualized by BF-TEM at two different magnifications.

Thermogravimetric analysis (TGA) of Pt pyramidal nanoparticles on Vulcan XC72 carbon

The mass of Pt NPs dispersed on carbon was determined via thermogravimetric analysis (TGA Q500 V20.13 Build 39). Before the analysis, the sample was lyophilized. The amount of Pt on carbon was measured comparing the results with the same quantity of a sample composed of only amorphous carbon. To start the measurement, a ceramic crucible was previously cleaned in aqua regia and, thereafter, thoroughly rinsed with ultrapure water and dried. Measurements were performed under air flow at a rate of 50.0 mL·min^{-1}, starting from 30 °C and increasing the temperature with a rate of 10 °C/min up to 600 °C. The mass percentage of these Pt pyramidal NPs on the carbon was measured by thermogravimetric analysis (TGA) measurements (Figure S12).

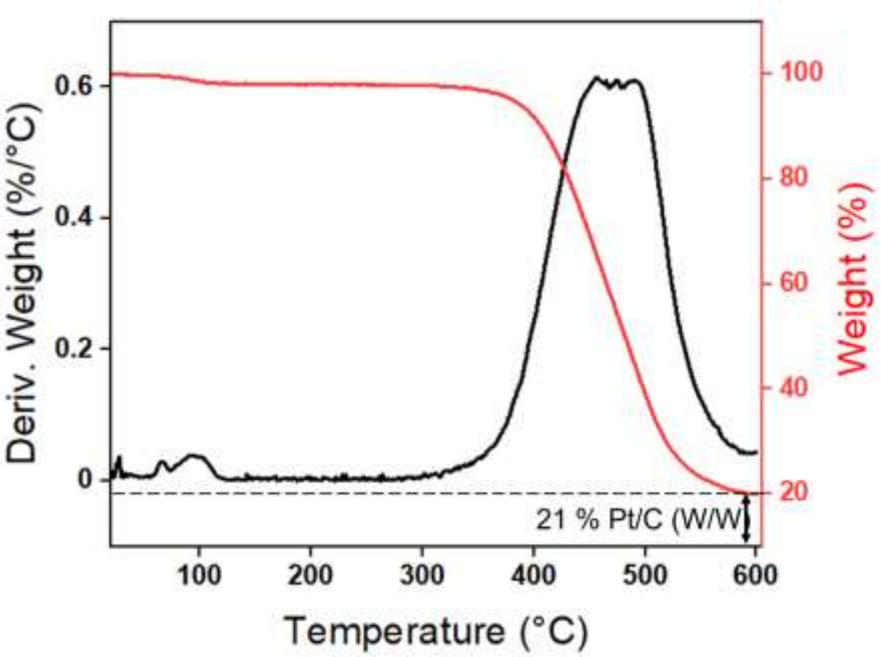

Figure S12. Thermogravimetric analysis (TGA) of the Pt pyramidal nanoparticles on Vulcan XC72 carbon, proving a mass percentage equal to 21%.

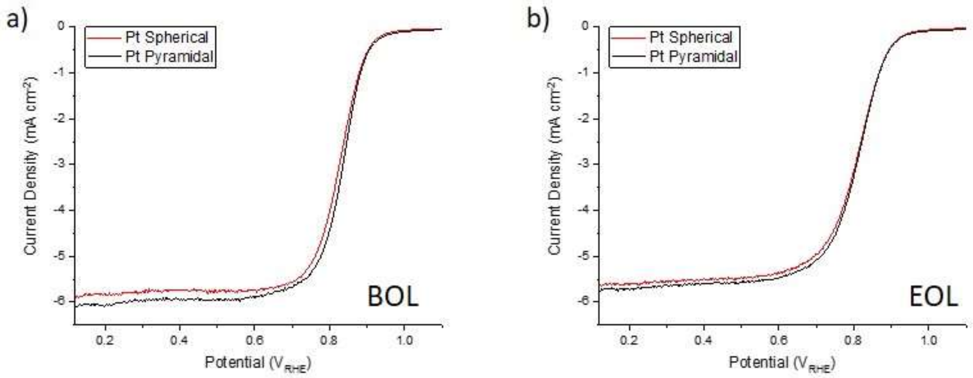

Figure S13. Linear sweep voltammograms for ORR in O_2 saturated 0.1 M $HClO_4$. (a) Beginning of life and (b) end of life ORR activity, measured before and after the 0.6 -1 V_{RHE} AST, for pyramidal and spherical Pt catalysts. Rotation rate 1600 rpm, scan rate 10 mV s^{-1}.

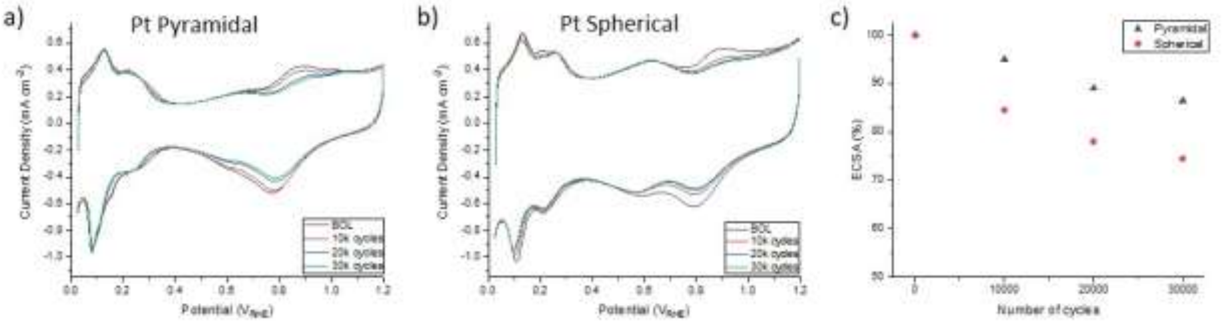

Figure S14. Cyclic voltammograms for (a) pyramidal and (b) spherical Pt catalysts, measured during the 0.6 - 1 V_{RHE} accelerated stress test, with a 20 mV s^{-1} scan rate. (c) Associated ECSA percentage change for the two catalysts.

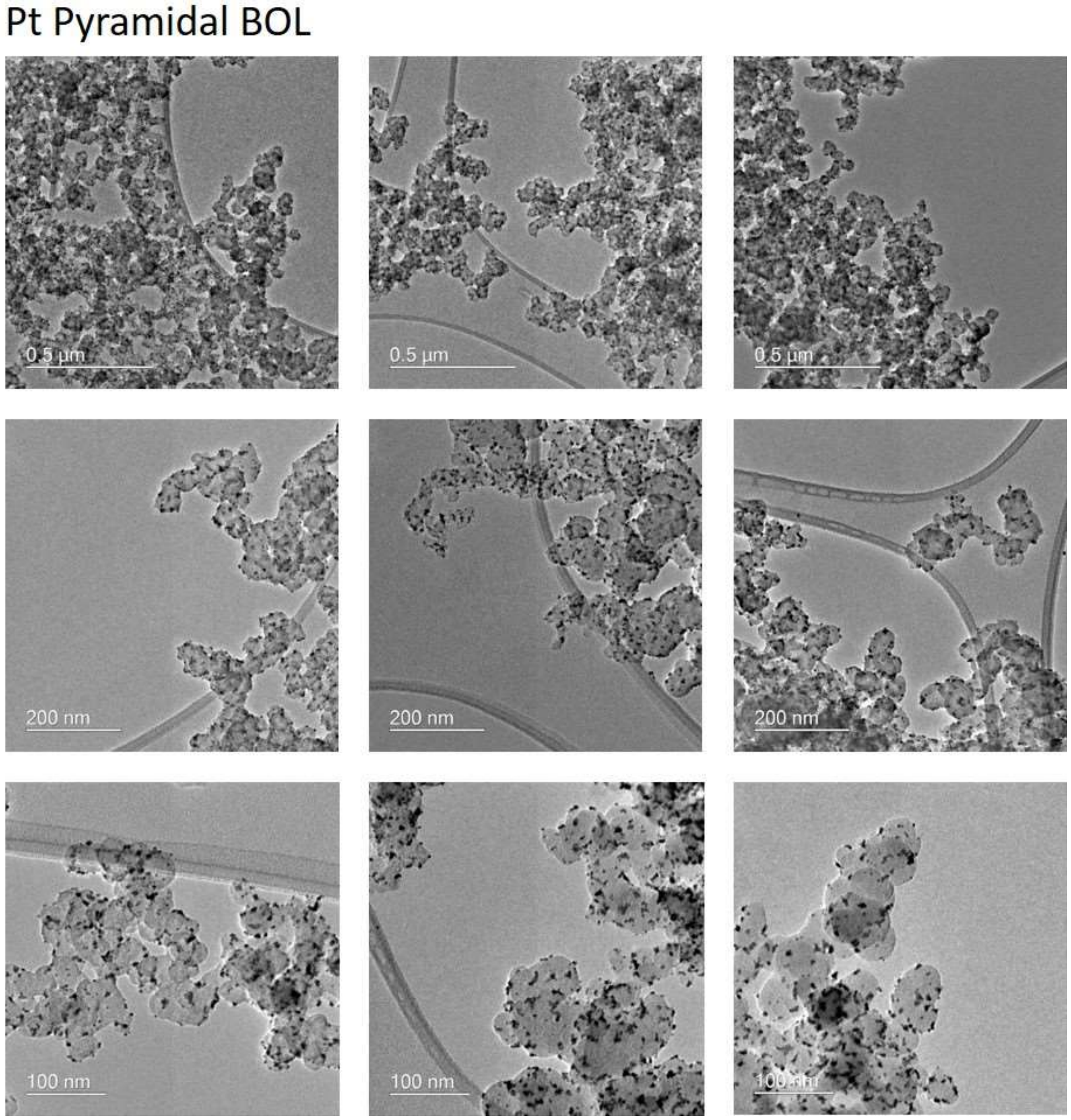

Figure S15. Beginning of life TEM images, at varying length scales, for the pyramidal Pt catalyst.

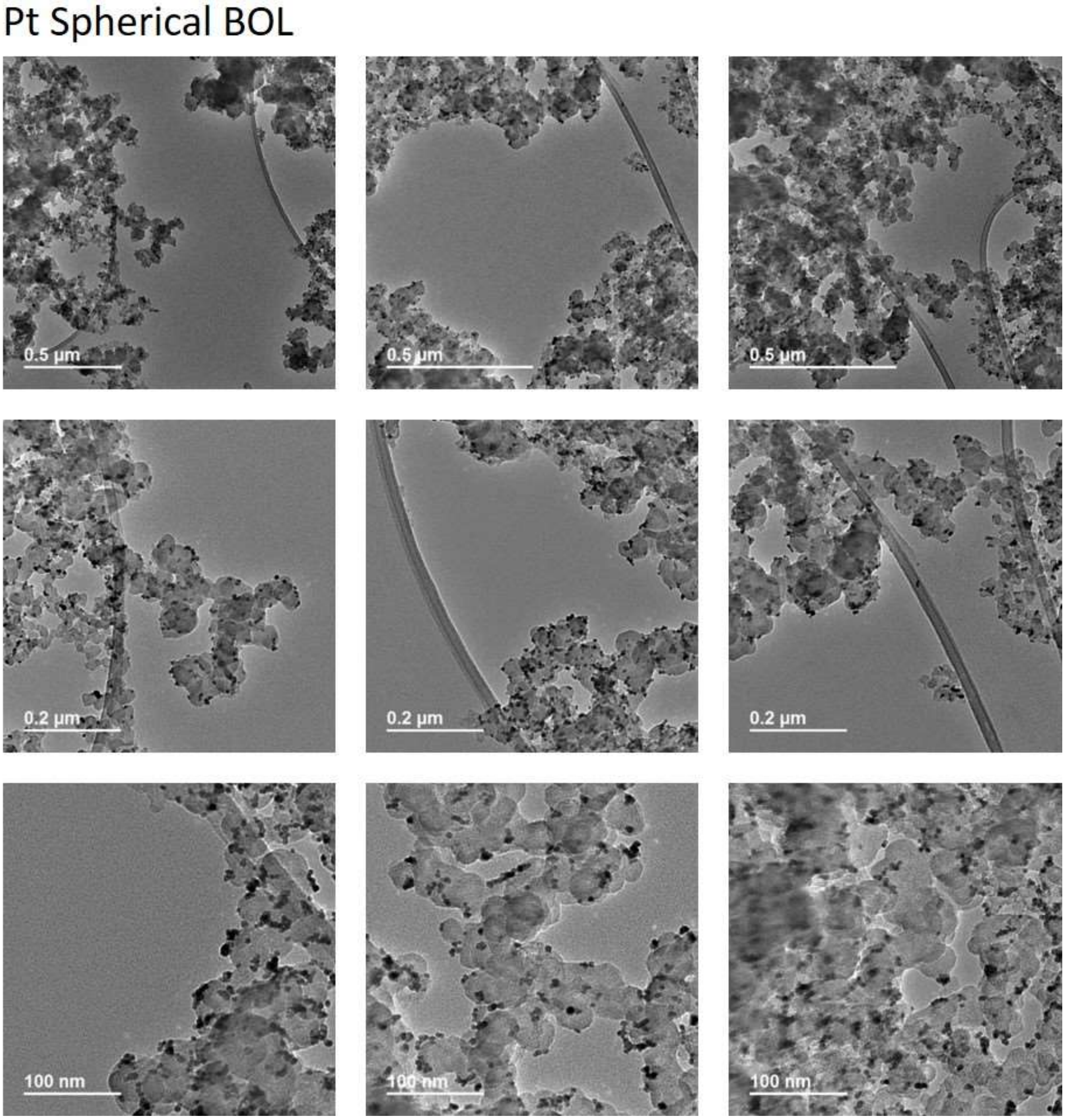

Figure S16. Beginning of life TEM images, at varying length scales, for the spherical Pt catalyst.

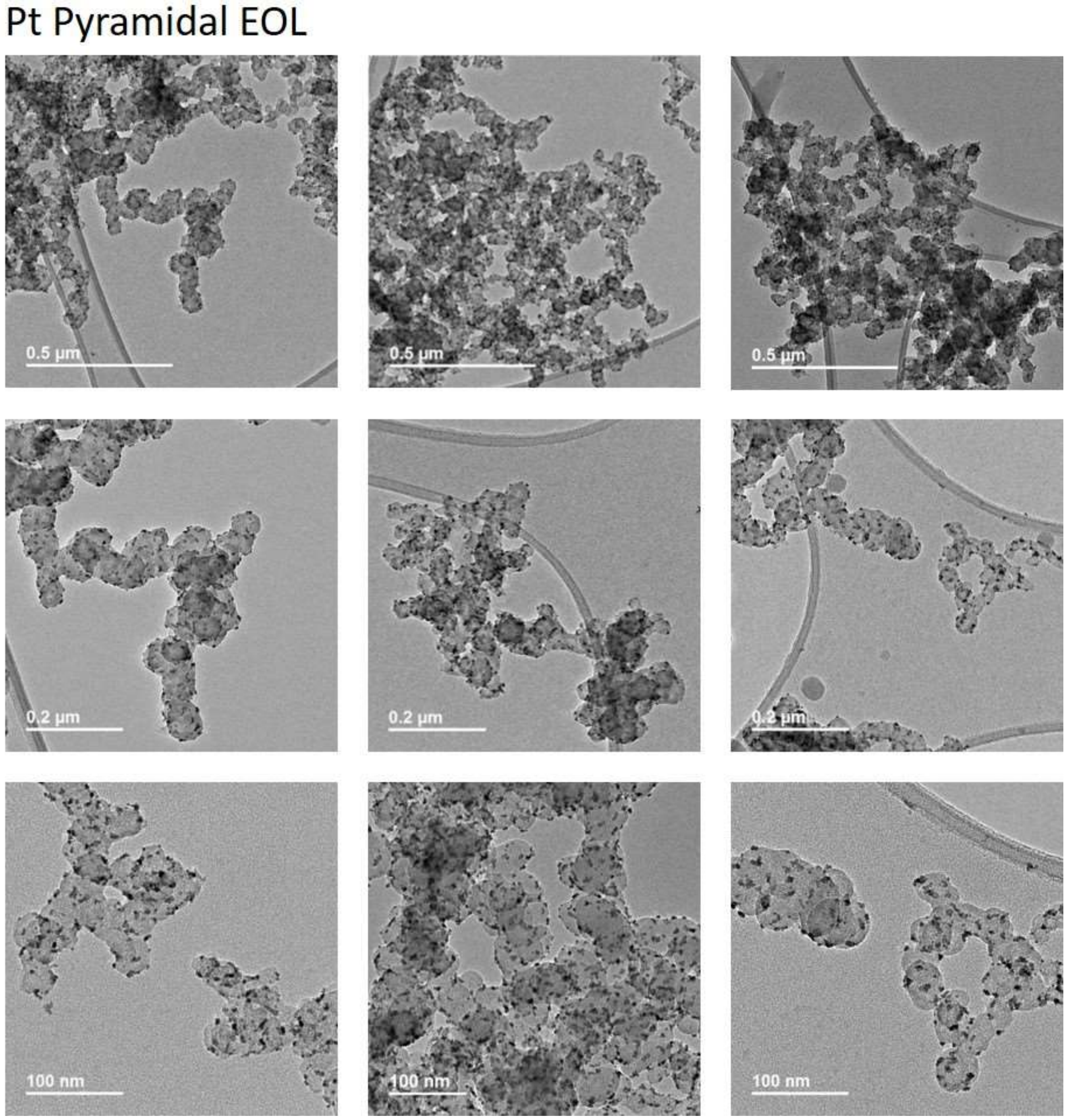

Figure S17. End of life TEM images, at varying length scales, for the pyramidal Pt catalyst, collected after the 0.6-1 V_{RHE} accelerated stress test.

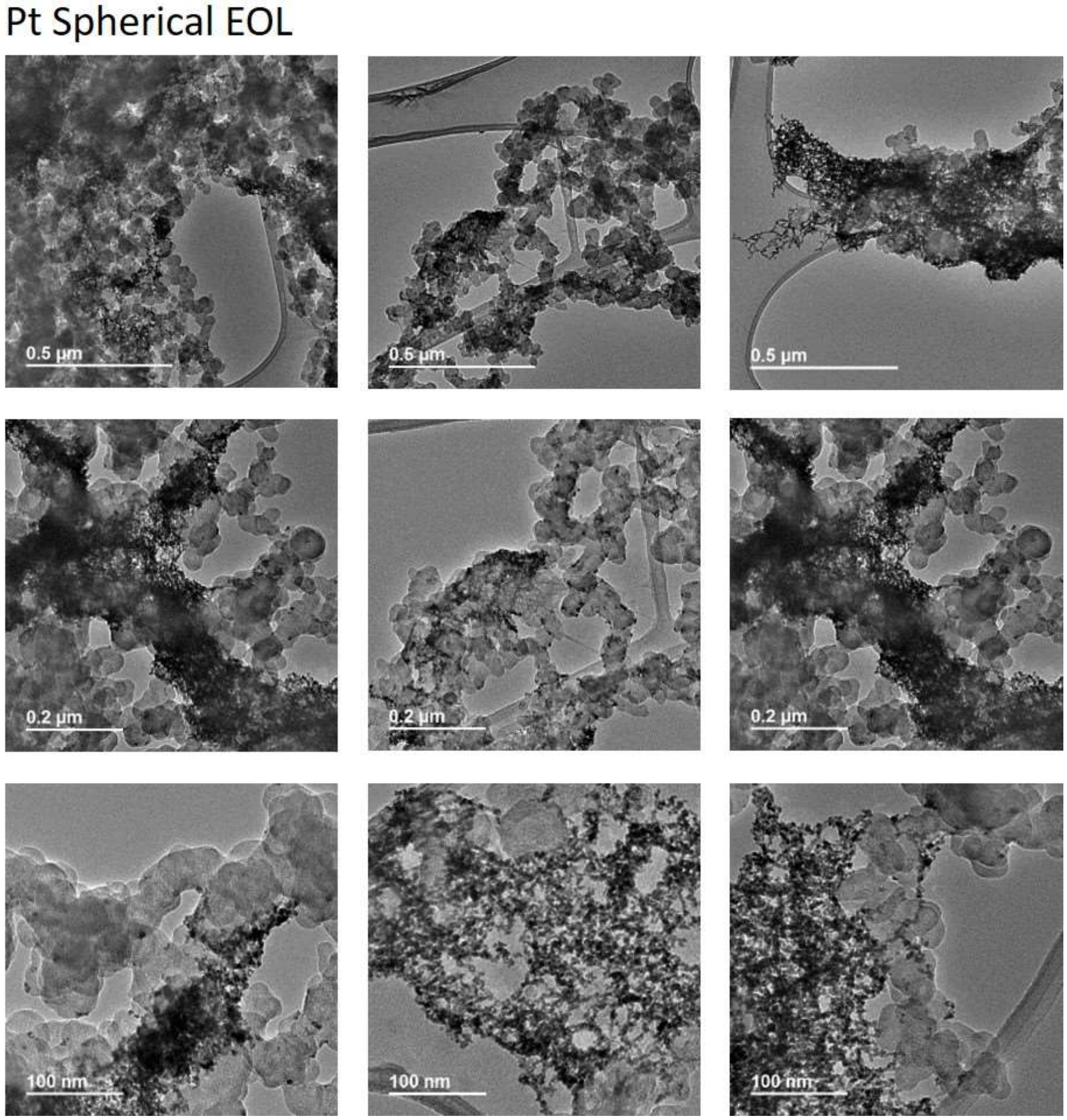

Figure S18. End of life TEM images, at varying length scales, for the spherical Pt catalyst, collected after the 0.6-1 V_{RHE} accelerated stress test.

Pt Pyramidal EOL

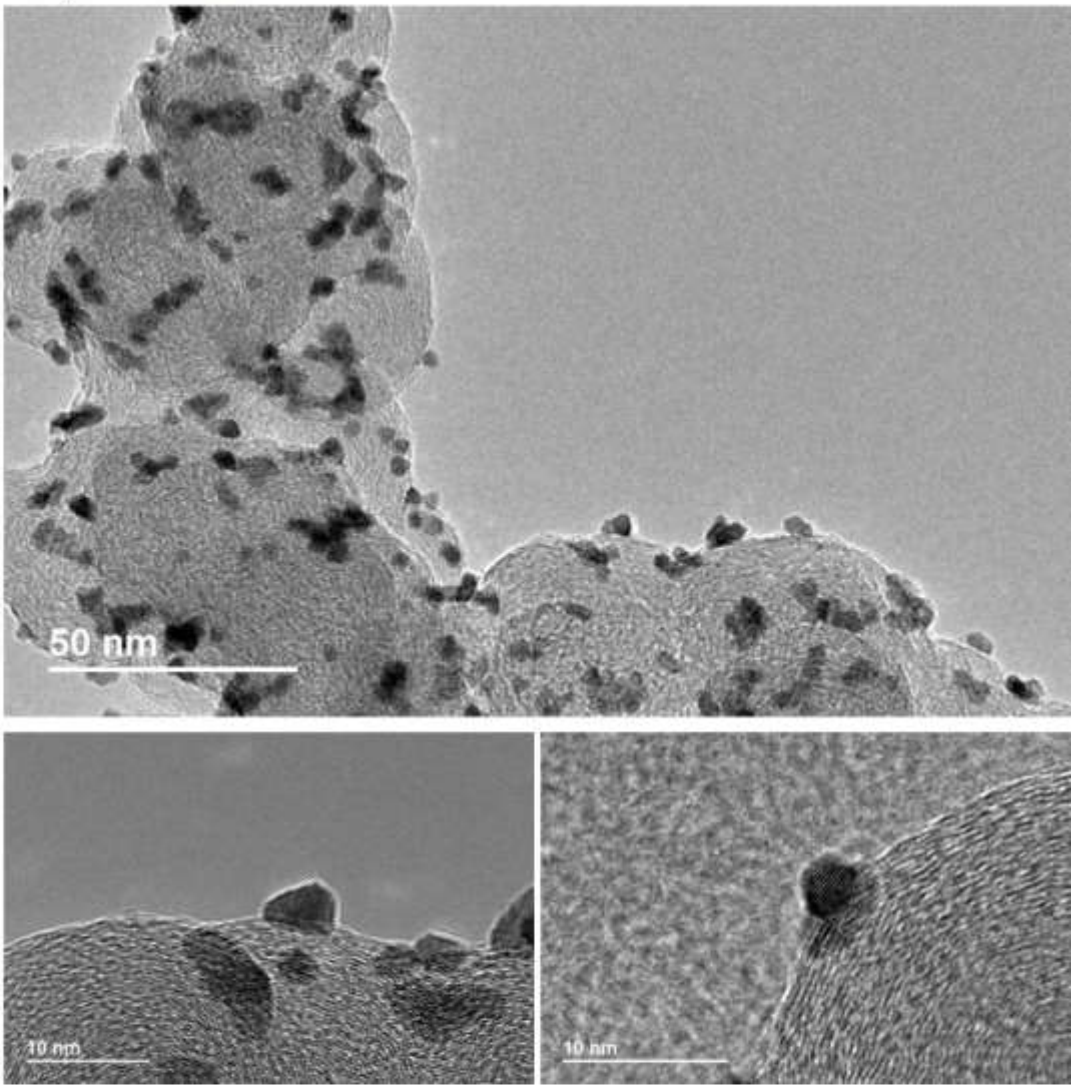

Figure S19. High magnification TEM images for the pyramidal Pt catalyst, collected after 30,000 cycles of the 0.6-1 V_{RHE} accelerated stress test.

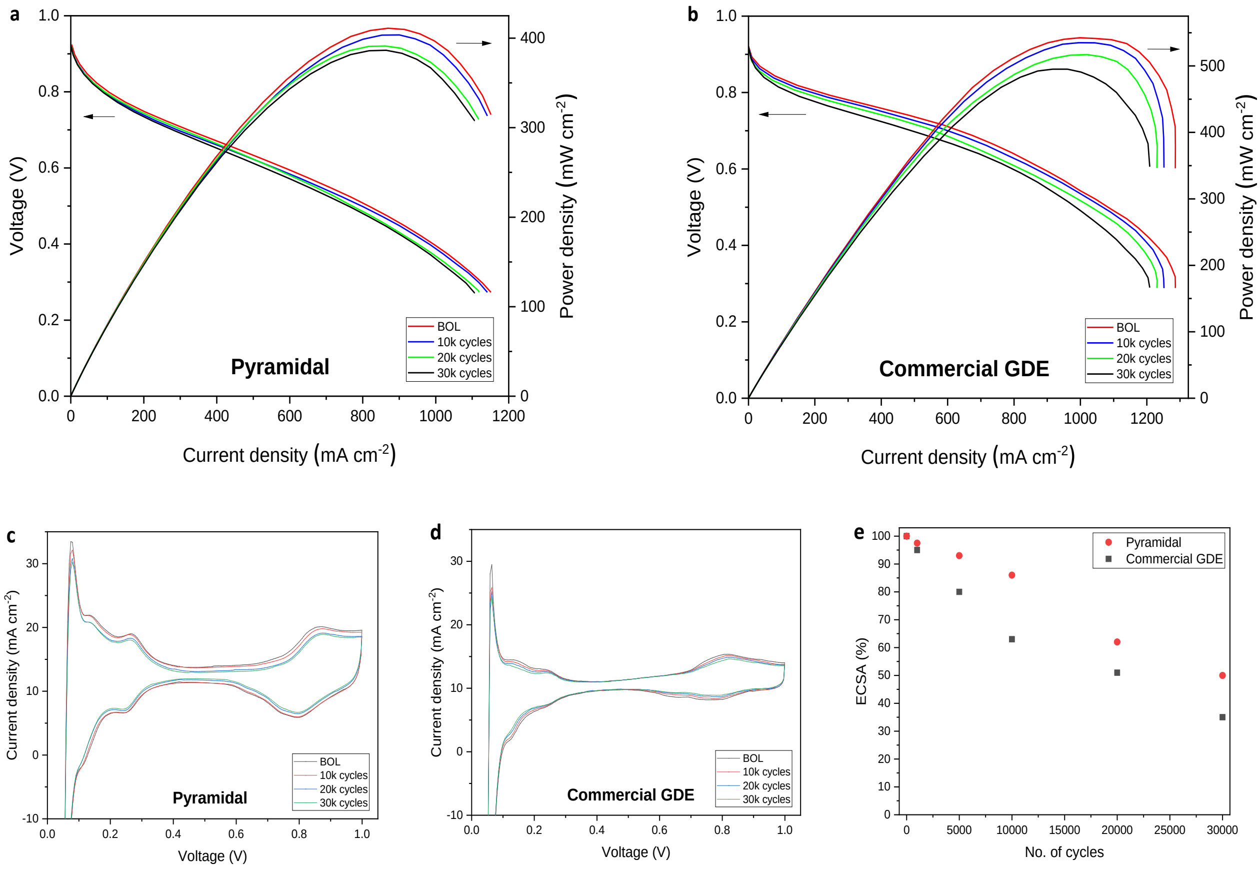

Figure S20. Electrocatalytic Fuel Cell characterisation. Full PEMFC polarisation curves at the BOL and after 10 – 30k H_2/air AST cycles[10] for (a) pyramidal and (b) commercial GDEs, with a cathode loading of 0.1 mg_{Pt} cm^{-2}. Cyclic voltammograms for (c) pyramidal and (d) commercial GDEs, measured during the enhanced accelerated stress test, with a 20 mV s^{-1} scan rate. (e) Associated ECSA percentage change for the two MEAs.

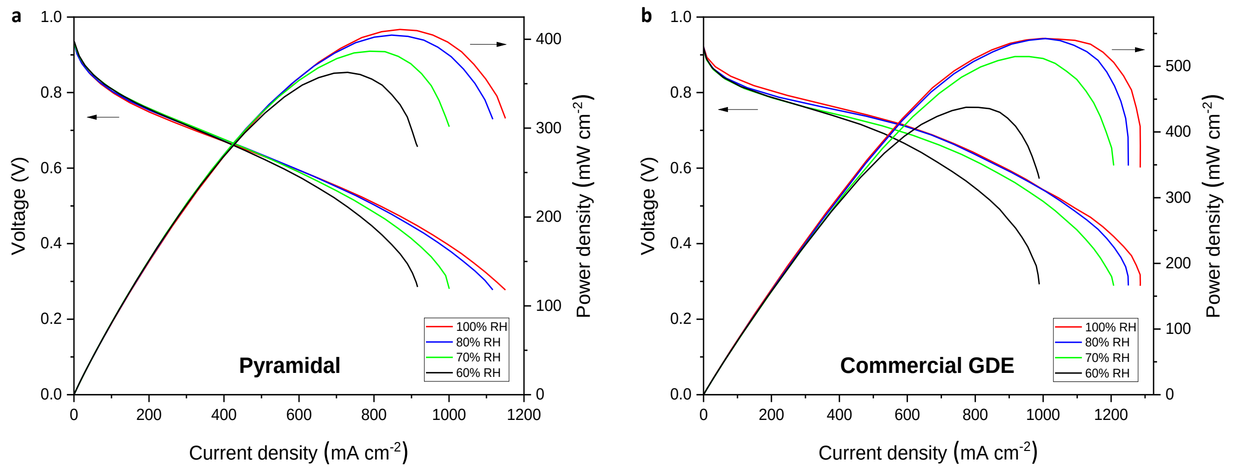

Figure S21. Electrocatalytic Fuel Cell characterisation. Polarisation curves at different relative humidity, from 60% to 100% RH, for (a) pyramidal and (b) commercial GDEs. Both with a cathode

loading of 0.1 mg_{Pt} cm^{-2}. Tests were performed using H_2/air stoicheometry of 1.5/2 at a cell temperature of 80 ˚C.

References

1. Hornberger, E.; Mastronardi, V.; Brescia, R.; Pompa, P. P.; Klingenhof, M.; Dionigi, F.; Moglianetti, M.; Strasser, P., Seed-Mediated Synthesis and Catalytic ORR Reactivity of Facet-Stable, Monodisperse Platinum Nano-Octahedra. *ACS Applied Energy Materials* **2021,** *4* (9), 9542-9552.
2. Dionigi, F.; Weber, C. C.; Primbs, M.; Gocyla, M.; Bonastre, A. M.; Spöri, C.; Schmies, H.; Hornberger, E.; Kühl, S.; Drnec, J.; Heggen, M.; Sharman, J.; Dunin-Borkowski, R. E.; Strasser, P., Controlling Near-Surface Ni Composition in Octahedral PtNi(Mo) Nanoparticles by Mo Doping for a Highly Active Oxygen Reduction Reaction Catalyst. *Nano Lett.* **2019,** *19* (10), 6876-6885.
3. Hasché, F.; Oezaslan, M.; Strasser, P., Activity, Structure and Degradation of Dealloyed PtNi Nanoparticle Electrocatalyst for the Oxygen Reduction Reaction in PEMFC. *J. Electrochem. Soc.* **2011,** *159* (1), B24-B33.
4. Lee, C.-T.; Yang, X.; Vara, M.; Gilroy, K. D.; Xia, Y., Water-Based Synthesis of Sub-10 nm Pt Octahedra and Their Performance towards the Oxygen Reduction Reaction. *ChemNanoMat* **2017,** *3* (12), 879-884.
5. Bu, L.; Feng, Y.; Yao, J.; Guo, S.; Guo, J.; Huang, X., Facet and dimensionality control of Pt nanostructures for efficient oxygen reduction and methanol oxidation electrocatalysts. *Nano Research* **2016,** *9* (9), 2811-2821.
6. Wang, X.; Figueroa-Cosme, L.; Yang, X.; Luo, M.; Liu, J.; Xie, Z.; Xia, Y., Pt-Based Icosahedral Nanocages: Using a Combination of {111} Facets, Twin Defects, and Ultrathin Walls to Greatly Enhance Their Activity toward Oxygen Reduction. *Nano Lett.* **2016,** *16* (2), 1467-1471.
7. Ahn, C.-Y.; Park, J. E.; Kim, S.; Kim, O.-H.; Hwang, W.; Her, M.; Kang, S. Y.; Park, S.; Kwon, O. J.; Park, H. S.; Cho, Y.-H.; Sung, Y.-E., Differences in the Electrochemical Performance of Pt-Based Catalysts Used for Polymer Electrolyte Membrane Fuel Cells in Liquid Half- and Full-Cells. *Chem. Rev. (Washington, DC, U. S.)* **2021,** *121* (24), 15075-15140.
8. Feng, Y.; Huang, B.; Yang, C.; Shao, Q.; Huang, X., Platinum Porous Nanosheets with High Surface Distortion and Pt Utilization for Enhanced Oxygen Reduction Catalysis. *Adv. Funct. Mater.* **2019,** *29* (45), 1904429.
9. Liu, H.; Zhong, P.; Liu, K.; Han, L.; Zheng, H.; Yin, Y.; Gao, C., Synthesis of ultrathin platinum nanoplates for enhanced oxygen reduction activity. *Chem. Sci.* **2018,** *9* (2), 398-404.
10. Zhang, H.; Osmieri, L.; Park, J. H.; Chung, H. T.; Cullen, D. A.; Neyerlin, K. C.; Myers, D. J.; Zelenay, P., Standardized protocols for evaluating platinum group metal-free oxygen reduction reaction electrocatalysts in polymer electrolyte fuel cells. *Nature Catalysis* **2022,** *5* (5), 455-462.
